# Affibody Molecules Intended for Receptor-Mediated Transcytosis via the Transferrin Receptor

**DOI:** 10.3390/ph16070956

**Published:** 2023-07-03

**Authors:** Linnea Charlotta Hjelm, Hanna Lindberg, Stefan Ståhl, John Löfblom

**Affiliations:** Department of Protein Science, School of Engineering Sciences in Chemistry, Biotechnology and Health, KTH Royal Institute of Technology, 106 91 Stockholm, Sweden; lhjelm@kth.se (L.C.H.); hanli@kth.se (H.L.); ssta@kth.se (S.S.)

**Keywords:** affibody molecules, blood–brain barrier, transferrin receptor-1, receptor-mediated transcytosis, phage display, directed evolution

## Abstract

The development of biologics for diseases affecting the central nervous system has been less successful compared to other disease areas, in part due to the challenge of delivering drugs to the brain. The most well-investigated and successful strategy for increasing brain uptake of biological drugs is using receptor-mediated transcytosis over the blood–brain barrier and, in particular, targeting the transferrin receptor-1 (TfR). Here, affibody molecules are selected for TfR using phage display technology. The two most interesting candidates demonstrated binding to human TfR, cross-reactivity to the murine orthologue, non-competitive binding with human transferrin, and binding to TfR-expressing brain endothelial cell lines. Single amino acid mutagenesis of the affibody molecules revealed the binding contribution of individual residues and was used to develop second-generation variants with improved properties. The second-generation variants were further analyzed and showed an ability for transcytosis in an in vitro transwell assay. The new TfR-specific affibody molecules have the potential for the development of small brain shuttles for increasing the uptake of various compounds to the central nervous system and thus warrant further investigations.

## 1. Introduction

Antibodies and other biological drugs have had a profound impact on the treatment of many different diseases, such as different forms of cancers and auto-immune disorders [1,2]. However, for diseases of the central nervous system (CNS), the restricted uptake over the blood–brain barrier (BBB) [3,4] makes development of therapeutics and diagnostic tools more challenging.

The BBB protects the sensitive neuronal environment by substantially decreasing the paracellular diffusion of substances from blood. Various receptors and transporters such as the transferrin receptor-1, the insulin-like growth factor 1 receptor, the L-type amino acid transporter, and the glucose transporter isoform 1 control the transcytosis of molecules in and out between the brain and blood compartments [3,5,6]. Transfer can either be by adsorption [7,8,9,10], passive or active transportation [5], or receptor-mediated transcytosis (RMT) [5]. It has been shown that about 0.1–0.2% of systemically administered antibodies pass into the CSF [11], which is potentially too low to achieve efficient disease-modifying effects [12]. Therefore, efforts have been made to find a transportation system across the BBB for biological drugs [5,13,14,15,16,17]. One of the more well-studied approaches is RMT via the transferrin receptor-1 (TfR), which naturally transports transferrin (Tf) across the BBB to the neuronal environment [7,18,19]. An example is the anti-murine TfR-specific 8D3 shuttle antibody [20], which has shown up to 80-fold increased uptake to the brain in rodent models [21]. For antibodies targeting TfR, several parameters have to be considered, such as affinity [22], valency [21,23], and antibody effector functions to minimize potential adverse effects [24].

In addition to antibodies and antibody derivatives [17,24,25], shuttles based on modified fragment crystallizable (Fc) from antibodies [16] and alternative scaffolds are emerging. The alternative scaffolds include a variety of protein structures, such as VNARs [26] and cysteine-dense peptides [27]. With a monovalent format and lack of Fc, they might potentially circumvent some limitations and safety liabilities previously seen for bivalent antibodies, such as harmful immune responses caused by effector function and TfR receptor depletion [21,28,29,30]. Moreover, the small size of alternative scaffolds might benefit biodistribution and targeting due to more rapid diffusion in the brain parenchyma, as has been shown for single-chain variable fragments (scFv) when compared to full-length antibodies [31].

Affibody molecules are small (6.5 kDa) affinity proteins that fold into a three-helical structure and typically demonstrate high thermal stability, high solubility, and efficient and complete refolding after denaturation [32]. Production is efficient in prokaryotic *Escherichia coli,* and the small size allows for production by solid-phase chemical peptide synthesis, enabling the straightforward incorporation of unnatural amino acids [32,33]. Affibodies with new specificities are generated by directed evolution (e.g., phage and bacterial display [32,34,35]), and binders to over 60 different targets have been reported in the literature [32]. The most advanced affibody molecule in clinical testing is izokibep, with a femtomolar affinity for interleukin 17-A (IL17-A) [32,36]. Results from phase I and II clinical trials show high efficacy in various IL17-driven auto-immune disorders and a good safety profile [36,37]. Selections from affibody libraries against intrinsically disordered neurodegenerative peptides have resulted in dimeric variants of the affibody scaffold with an unusual three-dimensional structure and mode of binding. The atypical dimeric binders are denoted sequestrins [38], and an example is a sequestrin (Z_SYM73_) targeting amyloid beta with subnanomolar affinity [39]. In (APP)/PS1 transgenic AD animal models, Z_SYM73_ restored cognitive function, inhibited amyloid beta aggregation, and eliminated neurotoxic effects [39]. Compared to antibodies, the small size of affibodies and sequestrins results in improved tissue penetration [19], but transportation across the BBB is still limited, corresponding to bioavailability of around 0.1–0.2% in CSF [39]. In a follow-up study, Z_SYM73_ was therefore fused to an scFv(8D3) brain shuttle, resulting in a 9-fold increase in CSF bioavailability [40].

Encouraged by the results, affibody molecules were here selected against TfR using phage display technology. Two of the binders demonstrated cross-reactivity to both human and murine TfR and displayed non-competitive binding with human Tf. The two candidates were further refined by single amino acid mutagenesis and evaluated in terms of different characteristics, such as thermal stability and cell binding. Finally, the top candidates were investigated for their ability of RMT using TfR-expressing brain endothelial cell lines in a recombinant silk-based in vitro assay where an increased uptake to the basal side for the TfR-specific affibodies was observed.

## 2. Results

### 2.1. Phage Display Selections

**Figure 1:**
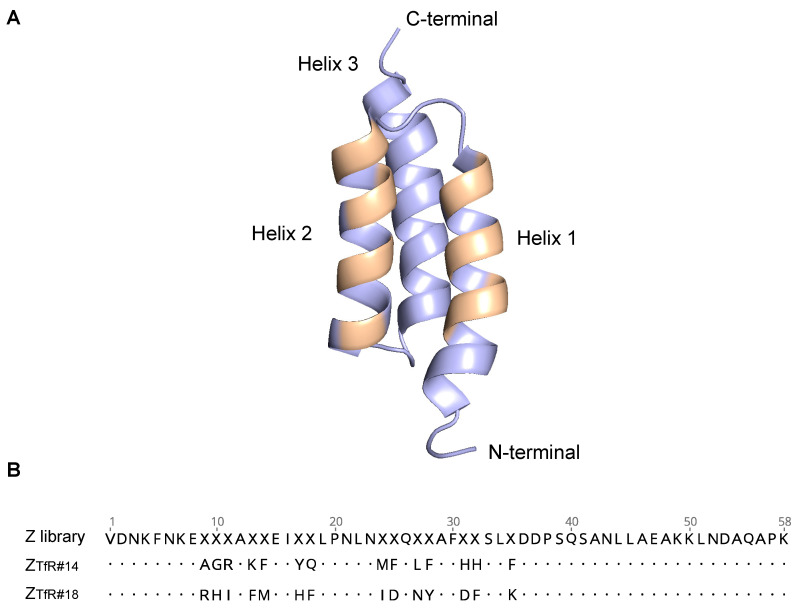
(**A**) Affibody structure with 14 randomized positions (beige) in helices 1 and 2 (PDB:2B89). (**B**) Z library sequence with randomized positions marked with “X” aligned to sequences for Z_TfR#14_ and Z_TfR#18_.

Affibody molecules were selected against the extracellular domain of recombinant human and murine transferrin receptors from an M13 filamentous phage library containing 3 × 10^10^ affibody molecules. The library was designed to include 14 surface-located randomized positions distributed over helices 1 and 2 (Figure 1A). Five rounds of panning of the library were performed in different tracks against human TfR, murine TfR, or a cross-selection strategy with alternating human and murine TfR (Figure S1). Target concentrations were gradually decreased from 100 to 12.5 nM (or 40 nM in cross-selection tracks) in the final rounds (Figure S1). Candidate binders were analyzed by phage ELISA for binding to the receptors, and 174 colonies from the last or second last cycle of panning were subjected to DNA sequencing.

### 2.2. Production of His_6_-Tagged and ABD-Fused Affibody Molecules

Nineteen different affibody molecules from the selections (Figure S2) were produced in both a His_6_-Z_TfR_-ABD and a Z_TfR_-His_6_ format. Affibody molecules can be fused to an albumin-binding domain (ABD) to prolong their half-life in circulation by interaction with human serum albumin (HSA) [36,41,42]. The production indicated a higher yield from the smaller format compared to the ABD-fusions (Tables S1 and S2). Molecular weight was verified by MS and correlated well with expected values (Tables S1 and S2). The purity was verified by SDS-PAGE (Figures S3 and S4), and all constructs showed the expected alpha-helical secondary structure content as measured using circular dichroism (CD) spectroscopy (Figure S5).

### 2.3. Flow Cytometry Analysis of Z_TfR_ Candidates Aimed to Bind to Murine and Human Cells

**Figure 2:**
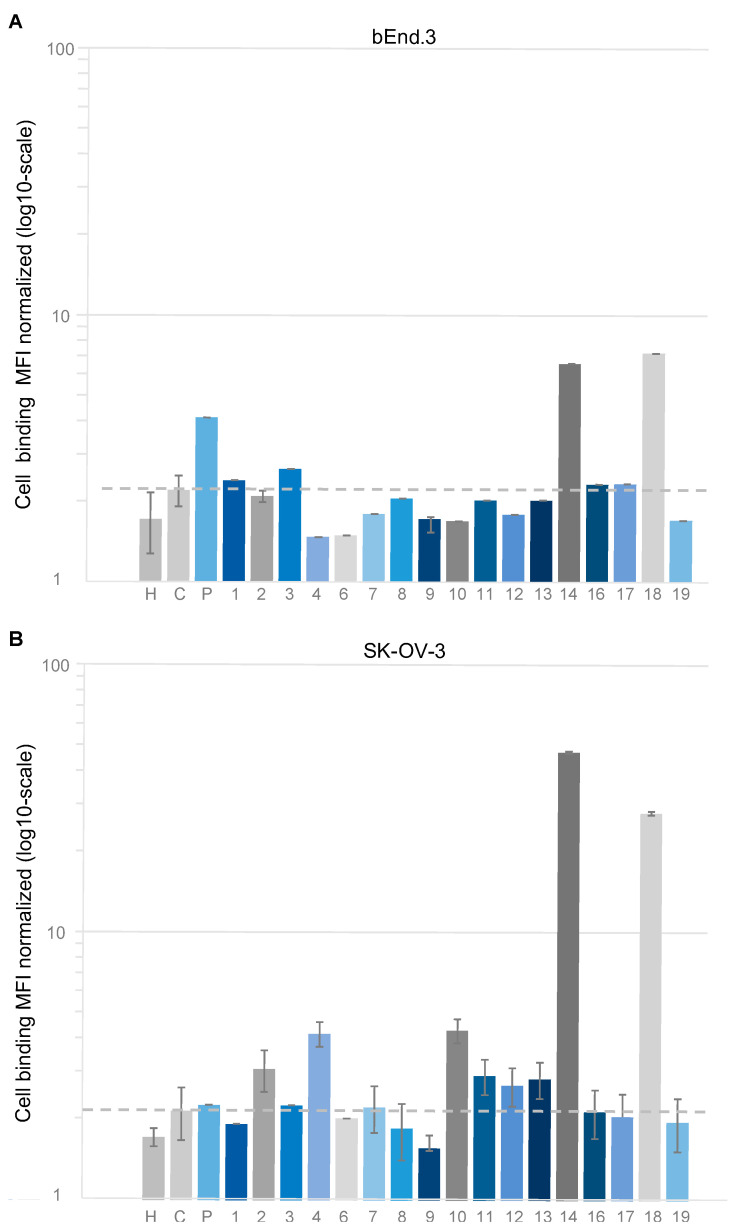
Analysis by flow cytometry of Z_TfR_-ABD constructs at 1 μM on (**A**) murine hTfR expressing bEnd.3 cells and (**B**) human TfR-expressing SK-OV-3 cells. The bar chart shows the MFI (mean fluorescent intensity) of TfR-binding, and signals are normalized to blank cells for respective cell lines. The first bar in both groups is negative control with cells incubated only with a secondary reagent (*H). The dashed line represents the signal from the negative control affibody molecule (Ztaq-ABD) (*C). A construct scFv8D3-Z_SYM73_-ABD (*P) with an scFv fragment of the murine TfR-specific antibody 8D3 is included as a positive control. Values are given as mean ± s.d. based on *n* = 3 samples.

The affibody candidates in Z_TfR_-ABD format were analyzed using flow cytometry for binding to human SK-OV-3 and murine brain endothelial cells (bEnd.3) expressing human and mouse TfR (Figure 2). Two of the affibody molecules (Z_TfR#14_ and Z_TfR#18_) demonstrated binding to both human SK-OV-3 and murine bEnd.3 (Figure 2, Table S3). The two binders originated from the selection track with alternating mTfR and hTfR as targets (Figure S1). Z_TfR#14_ and Z_TfR#18_ showed low sequence homology, which is typically an indication of non-overlapping epitopes (Figure 1B).

**Figure 3:**
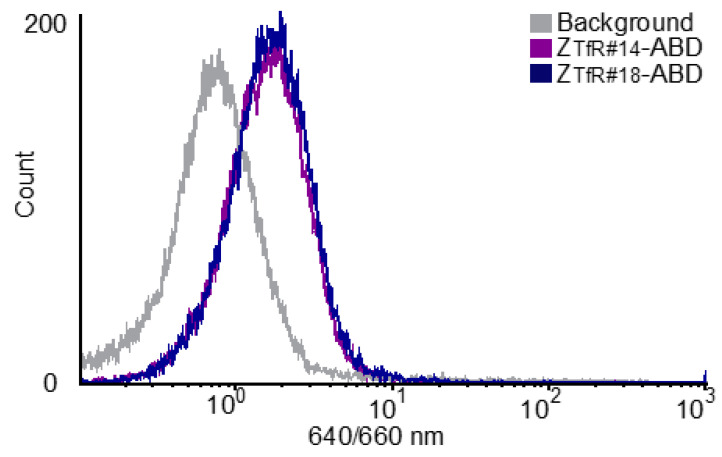
Binding to human brain endothelial cells. Flow cytometry showing binding to hCMEC/D3 cells for 300 nM of Z_TfR#14_-ABD (purple) and Z_TfR#18_-ABD (blue) with detection via HSA-647 at 640 nm excitation and 660 nm BP filter. In total, 20,000 cells were analyzed per sample.

Next, TfR-positive human SK-OV-3 and brain endothelial hCMEC/D3 cells were incubated with a dilution series of Z_TfR#14_ and Z_TfR#18,_ and binding was analyzed by flow cytometry, showing concentration-dependent signal (Figure S6 and Figure 3).

Cell-specific binding was investigated by co-incubating the His_6_-Z_TfR_-ABD construct with Z_TfR_-His_6_ at different molar excess ratios, showing a substantial decrease in signal in comparison to no blocking (Table S4). Moreover, flow cytometry was also used for epitope binning by co-incubating the His_6_-Z_TfR#14_-ABD construct with Z_TfR#18_-His_6_ and vice versa, showing a negligible decrease in binding and thus confirming non-overlapping epitopes (Table S4).

**Figure 4:**
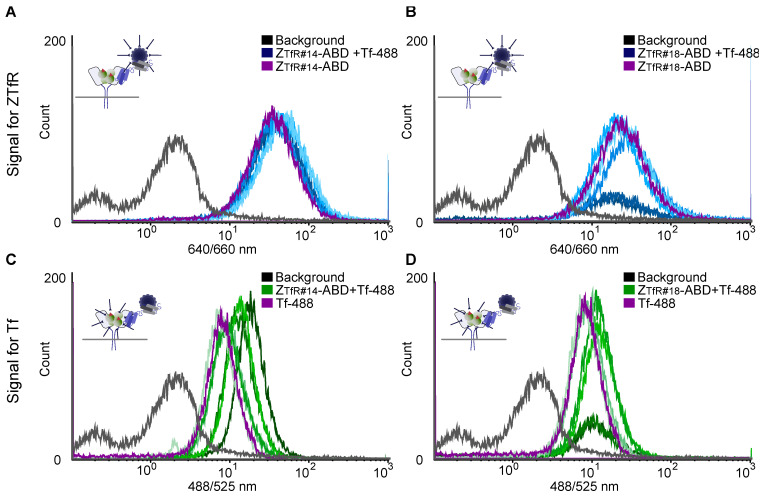
Flow cytometry of SK-OV-3 cells. (**A**,**B**) Fluorescence from cells corresponding to affibody-binding. Z_TfR#14_-ABD or Z_TfR#18_-ABD co-incubated with a different molar excess of transferrin-AF488 (Tf-488). Cells incubated with only affibody are shown in purple. Cells incubated with affibody and Tf-488 are shown in blue (higher molar excess in a darker shade). The Z_TfR_-ABD is detected by HSA-647 at 640/660 nm laser and filter. (**C**,**D**) Fluorescence from cells corresponding to Tf-488 binding. Z_TfR#14_-ABD or Z_TfR#18_-ABD co-incubated with a different molar excess of Tf-488. Cells incubated with only Tf-488 are shown in purple. Cells incubated with affibody and Tf-488 are shown in green (higher molar excess in a darker shade). Tf-488 is detected at 488/525 nm laser and filter. All samples are measured for 20,000 cells.

The high concentration of Tf in blood and results from previous studies demonstrating negative effects on reticulocyte count for transferrin-blocking agents in animal models [29] suggest that competitive binding with transferrin (Tf) is probably not optimal. Another epitope-binning experiment was thus performed, where the Z_TfR_ candidates #2, #4, #10, #14, and #18 in Z_TfR_-ABD format were pre-incubated with fluorescently labeled transferrin (Tf-488) prior to analysis of cell-binding. For clones #2, #4, and #10, the Tf-488 signal decreased after co-incubation, whereas no shift in signal was observed for co-incubation with Z_TfR#14_ and Z_TfR#18_ (Figure S7). Furthermore, the signal from Z_TfR#14_ and Z_TfR#18_ was maintained at a 5-fold molar excess of Tf (Figure 4). A non-TfR binding control affibody (Ztaq) [43] was included in the experiment, showing no effect on TfR binding (Table S5) and supports the indication that the TfR epitope is not shared between the Z_TfR#14_, Z_TfR#18_, and Tf.

### 2.4. pH-Dependent Binding to TfR Expressing Cells

**Figure 5:**
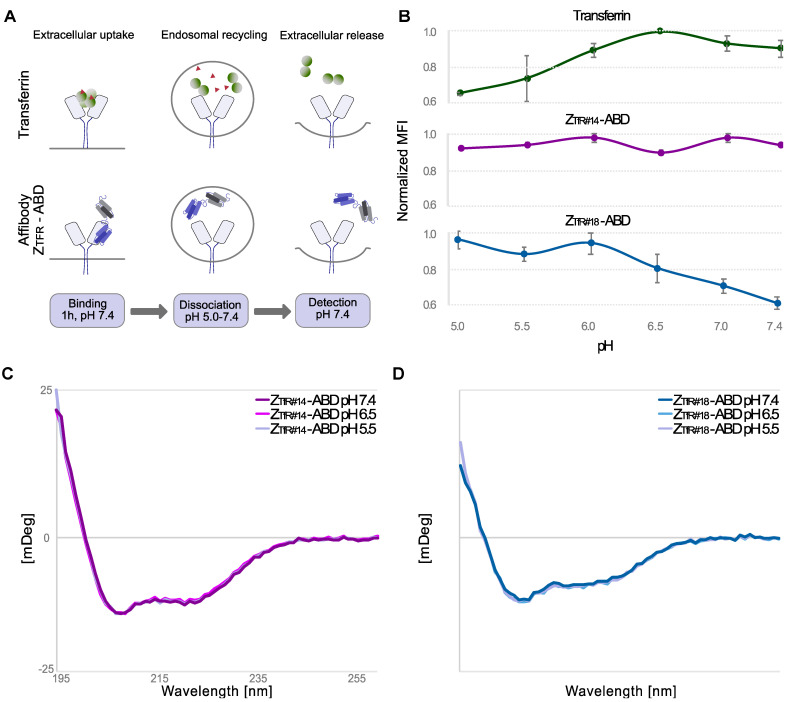
Evaluation for pH-dependent binding of the affibody constructs. (**A**) Schematic illustration of the experimental flow cytometric procedure. The sample is subjected to cells at physiological pH before washing and divided into different samples for dissociation at different pH for 30 min. The samples are finally washed at pH 7.4 and analyzed in the flow cytometer. Values are given as mean ± s.d. based on *n* = 3 samples. (**B**) The signal was obtained after incubation at different pH for Tf, Z_TfR#14_, and Z_TfR#18_. (**C**,**D**) Measurement of secondary structure by circular dichroism spectroscopy for Z_TfR#14_ and Z_TfR#18_ at pH 5.5, 6.5, and 7.4.

During transcytosis across the brain endothelial cells in the BBB, the pH of the endosomes containing TfR decreases to around 5.5 [18,19]. Thus, the dissociation of the affibodies from cells at different pH was investigated by employing a flow-cytometric assay similar to previously described studies [44] (Figure 5A). The analysis was performed at 4 °C to decrease internalization, and fluorescently labeled Tf (Tf-488) was included for comparison (Figure 5B). After incubation of binders with SK-OV-3 cells, cells were washed with buffers of different pH, followed by an analysis of cell-binding using flow cytometry. The pH-dependent binding of Tf–TfR was verified, shown as a decrease in signal for pH below 6.0 (Figure 5B). Interestingly, Z_TfR#14_ showed a pH-independent profile, and Z_TfR#18_ had opposite dependence compared to Tf, where the dissociation appeared slower at lower pH (Figure 5B). CD spectroscopy was used to verify secondary structure content, thermal stability, and refolding in the pH range (5.5–7.4) (Figure S8). The spectra measured at different pH were overlapping, indicating that the structure was similar and independent of pH in the given range (Figure 5C,D). The thermal melting point (T_m_) was estimated to be 56 °C for Z_TfR#14_ and 58 °C for Z_TfR#18,_ and spectra before and after the variable temperature measurements were overlapping, indicating full refolding (Figure S9).

### 2.5. Surface Plasmon Resonance Analysis of Binding between Z_TfR_ and TfR

The affinity of the Z_TfR#14_-ABD and Z_TfR#18_-ABD to recombinant TfR was estimated by surface plasmon resonance (SPR) using an immobilized extracellular domain of human and murine his-tagged TfR. Both affibody molecules showed a higher affinity for murine TfR with a K_D_ of 90 nM for Z_TfR#14_ and 170 nM for Z_TfR#18_. For human TfR, the K_D_ was 150 nM for Z_TfR#14_ and 710 nM for Z_TfR#18_ (Figure S9 and Table S6).

### 2.6. Single Amino Acid Mutagenesis of Z_TfR#14_ and Z_TfR#18_

Single amino acid mutagenesis was performed on Z_TfR#14_ and Z_TfR#18_ to investigate the contribution of individual residues in binding to TfR. The previously 14 randomized positions of respective affibodies were mutated to either histidine or the wildtype amino acid from the original IgG-binding Z domain [45]. The genes for the mutated affibody molecules were subcloned in fusion to a gene encoding an engineered albumin-binding domain (ABD) [41] into an expression vector for Adhesin Involved in Diffuse Adherence (AIDA) 1-mediated display on the surface of *E. coli* [34]. *E. coli* expressing the different mutants on the surface were analyzed by flow cytometry for assessment of TfR-binding, and the original binders (Z_TfR#14_ and Z_TfR#18_) were included for comparison. In addition to the analysis of TfR-binding, cells were also incubated with saturating concentrations of fluorescently labeled HSA to assess surface expression levels.

**Figure 6:**
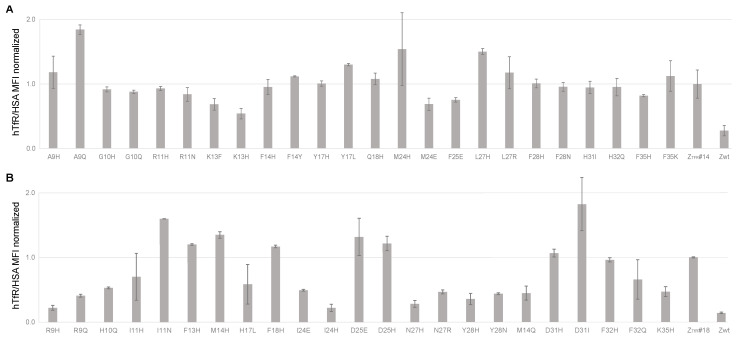
Mean fluorescence intensity (MFI) from flow cytometry on *E. coli* cells displaying single amino acid mutants of Z_TfR#14_ or Z_TfR#18_ in fusion to an albumin-binding domain. A negative control Z_wt_ [45] was included for the binding of hTfR to *E. coli*. Cells were incubated with labeled human TfR and fluorescently labeled HSA. MFI values are normalized by hTfR binding to expression levels (measured by HSA) and normalized with the signal for the original binder (Z_TfR#14_ or Z_TfR#18_). (**A**) Mutants of Z_TfR#14_ incubated with 100 nM hTfR, and (**B**) Mutants of Z_TfR#18_ incubated with 75 nM hTfR. Values are given as mean ± s.d. based on *n* = 2 samples.

For both affibody molecules, most mutations had a negative impact on the binding to TfR (Figure 6). However, two mutations with improved binding to TfR, for respective binder and with the introduction of histidine, were found, corresponding to A9H and L27H for Z_TfR#14,_ and M14H and I11N for Z_TfR#18_, although M14H seemed to have a negative effect on the cell surface expression (Figure 6 and Figure S10).

### 2.7. Characterization of Second-Generation Affibody Molecules Binding TfR

The four mutants (Z_TfR#14_A9H_, Z_TfR#14_L27H_, Z_TfR#18_I11N_, and Z_TfR#18_M14H_) that showed improved binding in the mutagenesis study were subcloned for expression of soluble affibody molecules in a (HE)_3_-Z_TfR_-cys format. In addition, two double mutants were generated and included in the study, corresponding to Z_TfR#14_A9H_L27H_ and Z_TfR#18_I11N_M14H_, as well as a control affibody with an affinity for the HER2 receptor [46] (Table S7, Figure S11). After purification, the C-terminal cysteine in the proteins was conjugated with biotin and FITC followed by verification of secondary structure content, molecular mass, thermal stability, and binding to TfR-positive cells (Figure S12, Tables S7 and S8).

The secondary structure content of the six mutants was similar to Z_TfR#14_ and Z_TfR#18,_ and thermal stability was either improved or similar (Table 1). However, Z_TfR#14_L27H_ and Z_TfR#18_I11N_ showed non-complete refolding after heat-induced denaturation. All six mutants demonstrated binding to both SK-OV-3 and brain endothelial bEnd.3 cells and the highest signals were observed for Z_TfR#14_L27H_ and Z_TfR#18_M14H_ (Figure S12). The pH-dependency of cell binding was similar for the mutants compared to Z_TfR#14_ and Z_TfR#18_ (Figure S13).

### 2.8. Flow Cytometric Endocytosis Assay

To study the potential endocytosis of the affibody constructs, a previously described flow cytometry-based assay was used [47]. In the assay, the pH sensitivity of FITC is exploited. One emission maximum (FL1 525 nm) of FITC is pH-dependent, whereas the other maximum (FL2 575 nm) is not. Dextran-FITC was added to bEnd.3 cells and incubated at different pH, followed by flow-cytometric analysis of the fluorescence in FL1 and FL2. The pH was plotted against the FL1/FL2 ratio for a standard curve (Figure S14). The FITC-labeled affibody molecules were thereafter incubated with bEnd.3 cells, followed by washing with acidic buffer (pH 5.0) and neutral (pH 7.0) to release remaining membrane-bound affibodies. After neutralization with buffer (pH 7.4), FL1 and FL2 fluorescence were analyzed using flow cytometry.

The TfR-specific BBB shuttle antibody 8D3 has previously been analyzed using the assay, revealing a pH of around 6, which corresponds to recycling TfR endosomes [18,19,47]. The obtained FL1/FL2 ratios for the affibodies corresponded to a pH of around 5–6 for Z_TfR#18_-derived variants and to a pH of around 6–7 for the Z_TfR#14_-derived variants (Table 2).

### 2.9. Transcytosis across a Murine In Vitro BBB Model

To study potential transcytosis across the BBB, a previously described in vitro assay was employed [47]. The assay is based on transwells with recombinant spider silk nanomembranes supporting confluent monolayers of brain endothelial cells. The FITC-labeled affibody molecules were added to the apical side of bEnd.3 cell membranes together with a fluorescently labeled antibody (IgG-AF647) that is used as an internal control for the barrier integrity of the membranes. The HER2-specific affibody (Z_HER2_) was included as a control. After 90 min, the media on both sides were collected, and the AF647 and FITC fluorescence were measured using a fluorescence spectrophotometer. The fluorescence was finally used to calculate the apparent permeability (p_app_) [47].

**Figure 7:**
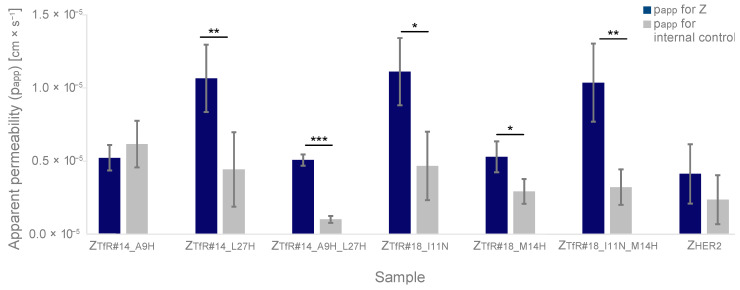
Apparent permeability (p_app_) for transcytosis of FITC labeled affibodies over a bEnd.3 cell barrier formed on recombinant silk membranes. Each sample is analyzed in at least triplicate and compared with the simultaneously added internal control in a two-sided students *t*-test (* *p*-value < 0.05, ** *p*-value < 0.01, *** *p*-value < 0.005). The Z_HER2_ control targets the HER2 receptor. Values are given as mean ± s.d. based on *n* = 3 samples.

All six TfR-specific affibody molecules except for Z_TfR#14___A9H_ showed higher apparent permeability than the internal control antibody (Table S9, Figure 7). Three of the variants also showed higher permeability compared to Z_HER2_, indicating transcytosis over TfR-positive bEnd.3 cells.

## 3. Discussion

The limited efficacy of many CNS-targeted biological drugs could potentially be improved by increased transportation across the BBB. Utilizing small alternative scaffolds (e.g., affibodies) as brain shuttles would potentially have an advantage over antibodies due to the faster biodistribution in the brain parenchyma after transcytosis. The smaller mass of the complex might additionally decrease potential systemic toxic effects from long serum half-life in the periphery [31,48]. RMT via the transferrin receptor-1 (TfR), which shuttles transferrin (Tf) and iron over the BBB to the brain [7,18,19], is one of the more well-investigated strategies. However, targeting the TfR receptor is still problematic as the receptor is abundantly present in other organs and cells in the body, resulting in a sink effect and potential toxicity issues, which have been observed in previous studies [40].

Herein, we selected TfR-specific affibody molecules using phage display technology. Nineteen candidates were selected from the output, and the two most promising binders were further characterized in vitro. Affinity, binding kinetics, epitope, valency, and pH-dependent binding are examples of properties that have been suggested in the literature to be important for RMT via TfR [23,29,49,50]. Moreover, cross-reactivity to TfR orthologues from model animals would facilitate future preclinical evaluation and clinical translation. When analyzing the two top candidates in flow cytometry on TfR-positive SKOV-3 cells, it was noted that the cell-binding signal decreased when labeling and analysis were performed at room temperature (data not shown), perhaps indicating internalization of the binders prior to the addition of secondary detection reagents.

The two candidates, Z_TfR#14_ and Z_TfR#18_, were cross reactive for murine and human TfR and bound to an epitope that was non-overlapping with the site for Tf. Avoiding the binding site of Tf is probably important as the high concentration of Tf in blood would otherwise greatly limit available receptors for RMT. Furthermore, blocking the interaction between Tf and TfR has previously been connected to toxicity in mice [29]. Using multi-color flow cytometry, we could show the simultaneous binding of Tf and respective affibody to cells at molar excess of either Tf or affibody. Thus, these affibodies will likely not be toxic due to decreased uptake of Tf.

It has been hypothesized and demonstrated for some TfR-specific antibodies in in vitro models that pH-dependent binding is important for transcytosis [49,51]. The idea is, in principle, to mimic the natural pH dependency in the range between pH 5.0 to 7.4, which is part of the iron transport mechanism via Tf and TfR. However, it should be noted that several TfR-specific brain shuttles without pH-dependent binding have been reported, and it is speculated that the importance of pH dependency is linked to TfR epitope and affinity, where both fast on rate and off rate are considered important [15,28,50,52]. The pH-dependent binding can, however, be of importance since certain epitopes, in combination with bivalent binders, are believed to cross-link the receptor during endocytosis, directing it to lysosomal degradation. Still, release by pH or by fast off rates after endocytosis probably has the potential to improve transcytosis [49,51]. The two candidates, Z_TfR#14_ and Z_TfR#18_, displayed different pH-dependent behavior, where Z_TfR#14_ was not affected by pH in the given range, and Z_TfR#18_ had a slower off rate from the receptor with lower pH.

Single amino acid mutagenesis was performed on the two candidates, where histidine or the Z_wt_ [45] amino acid was incorporated in the 14 previously randomized positions. The results from the mutagenesis study indicated the contribution of individual residues to the interaction with TfR. Moreover, a few mutants showed increased binding to TfR, and two single amino acid mutants for each affibody were analyzed further. The two mutations were also combined in double mutants to evaluate potential additive effects.

The new variants (Z_TfR#14_A9H_, Z_TfR#14_L27H_, Z_TfR#14_A9H_L27H,_ Z_TfR#18_I11N_, Z_TfR#18_M14H_, and Z_TfR#18I_11N_M14H_) were site-specifically conjugated to FITC and showed binding to both SK-OV-3 and brain endothelial bEnd.3 cells in flow cytometry. Internalization was analyzed using a fluorescence assay, exploiting the pH-dependency of FITC and indicating that the binders were located in a cell compartment with a pH of around 5–6 for the Z_TfR#18_-derived variants and around 6–7 for the Z_TfR#18_-derived variants. The results suggest that the Z_TfR#18_-derived variants are more efficiently internalized, although a pH of around five might indicate that it is partly directed to lysosomes, which is undesired.

Finally, the six affibodies were studied with a method where nanofibrillar membranes of recombinant silk seeded with brain endothelial cells are used as a simplified BBB model to assess active transcytosis. The results showed higher apparent permeability for three of the TfR-binders compared to negative control. As epitope, affinity, and internalization behavior only give indications about the capability of transcytosis over BBB, this assay is important as a predictor for the in vivo functionality.

The results are promising, and further optimization of the binders is warranted before studies on brain uptake in animal models.

## 4. Materials and Methods

### 4.1. Protein Labeling

Biotinylation of recombinant human and mouse transferrin receptor-1 (TfR: Sino Biological Inc., Bejing, China) was performed using a Biotin-XX Microscale Protein Labeling Kit (Invitrogen, Waltham, MA, USA) according to supplier’s recommendations. The concentration of the proteins was determined using absorbance at 280 nm.

### 4.2. Phage Display Selections

A combinatorial phage library of the Z domain with randomization in 14 positions was essentially prepared as described previously [53] (Figure 1). Selection and amplification were performed in phosphate-buffered saline with tween (PBST: 0.1% Tween-20) with bovine serum albumin (BSA: Saveen and Werner, Limhamn, Sweden) (PBSTB: 3 *w*/*v*% BSA) at room temperature, as described previously [35]. Prior to the first cycle of bio-panning, a negative selection of the library was conducted by incubating the library with streptavidin-coated magnetic beads. The remaining phage particles were panned against recombinant TfR for 1 h in five cycles with an increasing number of washes over the cycles. Different strategies for panning of binders were implemented based on recombinant human TfR, recombinant murine TfR, or cross-selection strategies. In the tracks where the target was kept constant, the concentration of the receptor was decreased from 100 nM in the first panning cycle to 12.5 nM in cycle five. In the cross-selection strategies, human and mouse receptor targets were altered in the five panning cycles, with target concentration lowered from 100 nM in the first two cycles to 40 nM in the last cycle (Figure S1). Selections were followed by DNA sequencing (Microsynth AG, Balgach, Switzerland) of 174 randomly picked colonies.

### 4.3. Production of Recombinant Affibody Molecules in E. coli

The affibody molecules selected for further characterization were produced with either a C-terminal hexahistidine (His_6_) tag or an N-terminal hexahistidine (His_6_) in combination with a C-terminal albumin binding domain (ABD) [41] for purification and characterization strategies. For His_6_-tagged proteins, the genetic sequences were subcloned into the pET26b(+) vector, introducing a C-terminal His_6_-tag and yielding the protein constructs [Z_TfR_]-His_6_. For ABD-fused proteins, the affibody genes were cloned into a pT7 vector introducing a C-terminal ABD_035_ molecule [41], yielding the final proteins His_6_-[Z_TfR_]-(G_4_S)_3_-ABD_035_. Transformation to *Escherichia coli* BL21STAR (DE3) cells (Thermo Scientific, Waltham, MA, USA) was performed by heat shock with the expression vectors, and colonies were sequence-verified by the Sanger sequencing (Microsynth AG, Balgach, Switzerland). For protein expression, cells were cultivated in tryptic soy broth with yeast extract (TSBY) media (Merck KGaA, Darmstadt, Germany) with 50 µg × mL^−1^ kanamycin at 37 °C with 150 rpm shaking. At OD_600_ of approximately 0.7 AU, protein expression was induced by the addition of Isopropyl β-D-1-thiogalactopyranoside (IPTG: Chemtronica, Stockholm, Sweden) to a final concentration of 1 mM. The cultures were incubated for approximately 18 h at 25 °C prior to harvest, and cells were lyzed by sonication with a Vibra-Cell VCX 130 sonicator (Sonics, Newtown, CT, USA). The affibody molecules were purified by immobilized metal affinity chromatography (IMAC) using a HisPur^TM^ Cobalt resin (Thermo Fisher Scientific, Waltham, MA, USA) with running buffer (47 mM Na_2_HPO_4_, 3 mM NaH_2_PO_4_, 300 mM NaCl, 15 mM imidazole, pH 7.4), and elution buffer supplemented with 150 mM imidazole. All eluted proteins were buffer exchanged on PD-10 columns (Cytiva, Marlborough, MA, USA) to phosphate-buffered saline (PBS). The protein concentrations were determined by a Pierce™ BCA Protein Assay Kit as per the manufacturer’s instructions (Thermo Scientific, Waltham, MA, USA). The molecular weight and purity of the proteins were confirmed by sodium dodecyl sulfate–polyacrylamide gel electrophoresis (SDS-PAGE) (NuPAGE Bis-Tris 4–12%, Invitrogen, Waltham, MA, USA) and MALDI mass spectrometry (MS) analysis using SCIEX 4200 MALDI-TOF instrument (SCIEX, Framingham, MA, USA).

### 4.4. Cultivation of TfR-Positive Cell Lines

Three cell lines were used to test for binding to TfR. The murine TfR-positive BBB cell line bEnd.3 (ATCC, United States) was cultivated in DMEM high glucose media (Thermo Fisher, Waltham, MA, USA) supplemented with 10% fetal bovine serum (FBS: Sigma-Aldrich/Merck KGaA, Darmstadt, Germany) at 37 °C with 5% CO_2_ until 80% confluency. The human TfR-positive SK-OV-3 (ATCC, Manassas, VA, USA) cell line was cultivated in McCoy media (Thermo Fisher, Waltham, MA, USA) supplemented with 10% FBS (Sigma-Aldrich/Merck KGaA, Darmstadt, Germany) under the same conditions. The human TfR-positive hCMEC/D3 (#SCC066, Merck KGaA, Darmstadt, Germany) endothelial brain cell line was cultivated in EndoGRO^TM^-MV Complete Media Kit (#SCME004, Merck KGaA, Darmstadt, Germany) supplemented with 1 ng × mL^−1^ fibroblast growth factor-2 (FGF-2) (#GF003, Merck KGaA, Darmstadt, Germany) in T75 flasks pre-coated with Collagen Type I, Rat Tail (#08-115, Merck KGaA, Darmstadt, Germany) in an atmosphere of 5% CO_2_ at 37 °C as by manufacturer’s instructions. To detach cells, TrypLE Express Gibco^TM^ (Thermo Fisher, Waltham, MA, USA) was added to cells in an incubator for 3–5 min. Recovered and washed cells were resuspended in ice-cold PBS supplemented with 1 *w*/*v*% BSA (PBSB) (Saveen and Werner, Limhamn, Sweden).

### 4.5. Flow Cytometry Analysis of Z_TfR_-Binding to Murine and Human Cells

A dilution series of each affibody in His_6_-[Z_TfR_]-ABD format was incubated with 100,000 human TfR-positive SK-OV-3 cells for 45 min, 15 rpm, 4 °C before washing twice with PBSB (1 *w*/*v*%). Fluorescently in-house labeled HSA (Sigma-Aldrich, St. Louis, MO, USA) with AF-647 (Thermo Scientific, Waltham, MA, USA) was used as a secondary reagent to detect binding and incubated with cells for 15 min on ice before washing away non-bound reagent. TfR expression on the cell lines was verified with fluorescently labeled human transferrin (Tf-488, Thermo Scientific, Waltham, MA, USA) and with ab18242 (Abcam, Cambridge, UK), as by manufacturer’s instructions. A Gallios^TM^ flow cytometer (Beckman Coulter, Brea, CA, USA) was used to analyze 20,000 events of each sample, and data were further analyzed in Kaluza (Version 2.1, Beckman Coulter, Brea, CA, USA). All experiments were performed in duplicates, and samples were normalized to blank cells for comparison between batches.

To analyze pH-dependent binding, SK-OV-3 cells were treated and analyzed as above, except for the following differences. His_6_-[Z_TfR_]-ABD and HSA-647 were preincubated for 20 min and then added to SK-OV-3 cells. After incubation, cells were washed in PBSB (1 *w*/*v*%) with different pHs in the range 5.0–7.4 for 30 min at 4 °C. All samples were analyzed in triplicate. Experimental set-up with inspiration from Neiveyans et al. [44].

To verify TfR-specific binding, a blocking experiment was performed by co-incubating the Z_TfR_-ABD construct with 5–10× molar excess of Z_TfR_-His_6_. The SK-OV-3 cells were treated and analyzed as above.

To analyze competitive binding with human transferrin, 25 μg × mL^−1^ (313 nM) fluorescently labeled transferrin (AF488) (#T13342, Invitrogen, Waltham, MA, USA) was pre-incubated with 100–600 nM of His_6_-[Z_TfR_]-ABD constructs prior to SK-OV-3 cell labeling and flow-cytometric analysis at 488/525 nm and 640/660 nm in triplicate. Additionally, 200 nM of respective His_6_-[Z_TfR_]-ABD construct was pre-incubated with 15–85 μg × mL^−1^ (186–1063 nM) of Tf-488 prior to SK-OV-3 cell labeling and flow-cytometric analysis at 488/525 nm and 640/660 nm in triplicate. Values were calculated as mean ± s.d.

### 4.6. Circular Dichroism Analysis for Secondary Structure

Circular dichroism (CD) spectroscopy was used to verify the secondary structure content of the affibody molecules in (HE)_3_-[Z_TfR_]-bio or His_6_-[Z_TfR_]-ABD format. CD spectra between 195–260 nm at 20 °C were recorded with a Chirascan system (Applied Photophysics, Leatherhead, UK) using a 1 mm High precision cell (110-1P-40 cuvettes, Hellma Analytics, Munich, Germany). Five scans were recorded for each protein sample at a concentration of 0.2 mg × mL^−1^ in PBS.

The thermal melting point for the affibody molecules was determined using a variable temperature measurement (VTM) at 221 nm with a temperature gradient of 5 °C or 1 °C per minute (depending on samples as indicated in results) with five readings at each temperature point. After cooling down to 20 °C, refolding was accessed by recording spectra and compared to the spectra before denaturation.

### 4.7. Biosensor Analysis of the Z_TfR_ and TfR Interaction of Both Murine and Human TfR

The affinity and kinetics of the interaction between the His_6_-[Z_TfR_]-ABD constructs and TfR of human and murine origin were analyzed using surface plasmon resonance (SPR) on a Biacore T200 system (Cytiva, Marlborough, MA, USA) at 25 °C with PBST (0.05% Tween-20) as running buffer. Approximately 1100 response units (RU) of hTfR-His_6_ and mTfR-His_6_ (Sino Biological Inc., Bejing, China) were immobilized on Series S CM5 chips (Cytiva, Marlborough, MA, USA) via amine coupling as per the manufacturer’s recommendations. The Z_TfR_ constructs were injected for 300 s at 30 μL × min^−1^ in a dilution series between 582–19.4 nM, in duplicate. Dissociation was monitored for 1000 s before regeneration with 10 mM HCl for 30 s, followed by a stabilization period of 60 s. Sensorgrams were double-referenced with the blank surface and a buffer injection of PBST and analyzed with Biacore evaluation software using 1:1 binding model.

### 4.8. Single Amino Acid Mutagenesis and E. coli Surface Display Binding Analysis

The Z_TfR#14_ and Z_TfR#18_ affibodies were mutated at 14 positions by introducing codons for histidine and the wildtype amino acid of the Z domain [45] at each position. Oligos encoding the mutated affibody genes (HT genparts, GenScript, Piscataway, NJ, USA) were subcloned to the *E. coli* display vector pBad2.2 [34] using Gibson assembly according to the supplier’s recommendations. Four fragments were assembled simultaneously in the Gibson assembly method (New England Biolabs, Ipswich, MA, USA) with a two times molar excess of the insert to pBad2.2 vector and transformed to BL21STAR (DE3) *E. coli* (Thermo Scientific, Waltham, MA, USA) by standard heat shock protocol. Colonies were sequence-verified by the Sanger sequencing (Eurofins Genomics, Ebersberg, Germany) and further transformed into electrocompetent *E. coli* JK321 [54].

### 4.9. Analysis of TfR-Binding Using E. coli Surface Display and Flow Cytometry

Transformed *E. coli* were grown in LB media at 37 °C until OD_600_ reached 0.5 AU before inducing with arabinose (0.6%) and incubation at 25 °C for 16 h protein expression. Cells expressing affibody molecules on the surface were incubated in 50 nM, 100 nM, and 200 nM of biotinylated hTfR (#H82E5, Acro Biosystems, Newark, DE, USA) for 45 min. After washing with PBS with 0.01% Pluronic F108 NF surfactant (PBSP), cells were incubated for 15 min on ice, with streptavidin phycoerythrin conjugate (SA-PE: Thermo Scientific, Waltham, MA, USA) and HSA-AF647 (in-house labeled). After washing, cells were analyzed on a Gallios™ Flow Cytometer system (Beckman Coulter, Brea, CA, USA), and data were analyzed in Kaluza (Version 2.1, Beckman Coulter, Brea, CA, USA) for forward/side scatter and relevant fluorophore signals (488/575 nm and 640/660 nm). Values were calculated as mean ± s.d based on duplicate readings.

### 4.10. Production and Labeling of Second-Generation Affibody Molecules

Selected clones from the mutagenesis study and a HER2-specific affibody Z_HER2:0342_ [46] as control were subcloned into the pET21a+-vector (Thermo Scientific, Waltham, MA, USA) modified to contain an N-terminal (HE)_3_-tag and C-terminal cystine using infusion cloning according to supplier’s recommendations (Takara, Kusatsu, Japan). The protein constructs were expressed in *E. coli* BL21STAR (DE3) as described above, with the change of carbenicillin (100 μg × mL^−1^). After sonication, the samples were treated at 70 °C for 10 min to precipitate host proteins. After incubation on ice for 20 min, samples were centrifuged for 20 min at 25,000× *g*. Samples were treated with 5 mM tris(2-carboxyethyl)phosphine (TCEP, Sigma-Aldrich/Merck KGaA, Darmstadt, Germany) for 30 min and thereafter purified using an ÄKTA start system (Cytiva, Marlborough, MA, USA) with a HisTrap crude column (Cytiva, Marlborough, MA, USA) using running buffer as described above (without imidazole) and elution buffer with 300 mM imidazole in a gradient elution (0–80%). Eluate fractions containing protein were buffer exchanged to PBS (pH 7.0) and concentrated to 2 mg × mL^−1^ before reducing with TCEP again for 30 min. The reduced proteins were thereafter conjugated to biotin (EZ-Link™ Maleimide-PEG2-Biotin, Thermo Scientific, Waltham, MA, USA) and Fluorescein-5-Maleimide (FITC) (Invitrogen, Waltham, MA, USA) as by manufacturer’s instructions. The secondary structure was verified by CD spectroscopy. Labeling efficacy and concentration after labeling was estimated by SCIEX 4200 MALDI-TOF instrument (SCIEX, Framingham, MA, USA), concertation by Pierce™ BCA Protein Assay Kit (Thermo Scientific, Waltham, MA, USA), and degree of labeling was estimated by using a NanoDrop spectrophotometer (NanoDrop Technologies, Wilmington, DE, USA). Purity was analyzed by SDS-PAGE (NuPAGE™ 4 to 12%, Bis-Tris, Invitrogen, Waltham, MA, USA).

### 4.11. Analysis of Endocytosis Using Flow Cytometry

Endocytosis was studied using a previously described flow cytometry method [47]. Briefly, bEnd.3 cells were resuspended in 0.1 mg × mL^−1^ Dextran-FITC (40 kDa Mw, Merck KGaA, Darmstadt, Germany) in ice-cold buffers ranging between pH 4.0–5.0 (25 mM Sodium Acetate, 25 mM NaCl, 125 mM KCl, 10 μM nigericin), pH 5.5–6.5 (25 mM MES, 25 mM NaCl, 125 mM KCl, 10 μM nigericin), or pH 7.0 (25 mM HEPES, 25 mM NaCl, 125 mM KCl, 10 μM nigericin), where nigericin was added just before use. Labeled cells were analyzed using flow cytometry for FL1 (488/525 nm) and FL2 (488/575 nm) to obtain a standard curve for pH. Each sample was analyzed in triplicate and normalized to blank cells without dextran. Values were calculated as mean ± s.d.

Next, cells were resuspended in ice-cold buffer pH 7.4 (25 mM HEPES, 25 mM NaCl, 125 mM KCl) buffer without nigericin and with 250 nM of respective FITC-labeled affibody molecule and incubated for 15 min, 15 rpm, at room temperature before spun down and washed once in a buffer of pH 5.0 and once at pH 7.0 (no nigericin) for 5 min at 15 rpm. The sample was resuspended in a pH 7.4 buffer and analyzed in the flow cytometer with the same settings as for the standard curve.

### 4.12. Transcytosis In Vitro Murine BBB Model

A previously described method based on recombinant spider silk nanomembranes with confluent monolayers of murine brain endothelial cells (bEnd.3) was used for the assessment of TfR-mediated transcytosis [47]. 500 nM of respective FITC-labeled affibody molecule together with an AF647-labeled internal-control IgG2a antibody was added in complete pre-warmed cell media to the silk membrane apical side with (n = 3) or without confluent bEnd.3 cell layer (n = 3) and placed into a well. Cell media was added to the basal side and incubated for 90 min (37 °C, 5% CO_2_). The apical and basal samples were aspired, and together with the starting sample, the fluorescent intensity was measured at 483-14/530-30 nm and 575-20/621-10 nm with 1200/3000 gain at 25 °C in a CLARIOStar Plus (BMG Labtech, Ortenberg, Germany). Values were averaged by technical and biological replicates, and signals from complete cell media were subtracted. Statistical analysis was performed in Microsoft Excel using a two-sided Student’s *t*-test with equal variance for the apparent permeability (*p_app_*, cm × s^−1^) calculated as by Equation ()1, where *A* is the surface area (cm^2^), *dQ*/*dt* the steady-state flux (mmol × s^−1^), *V_R_* is the volume of the receiver chamber (cm^3^) and *C*_0_ the initial concentration of the affibody (mM).

(1) $$p_{app}=\left(\frac{dQ}{dt}\right)\times \left(\frac{V_{R}}{A\times C_{0}}\right)$$

## 5. Conclusions

To conclude, we describe the development of affibodies targeting TfR by phage display technology. Two top candidates were evaluated in vitro and further improved by single amino acid substitutions, yielding a total of six new variants. The six affibodies were studied in terms of cell binding and transcytosis, demonstrating higher apparent permeability for three of the TfR-binders compared to a negative control. Although the affibody constructs evaluated herein show promising results, further optimization of the binders is likely warranted prior to studies on brain uptake in animal models.

## Figures and Tables

**Table 1:** Thermal melting point (T_m_) and refolding capability for biotinylated (HE)_3_-Z_TfR_-cys constructs.

Clone	T_m_ [°C]	Refolding Capability
(HE)_3_-Z_TfR#14_A9H_-biotin	64	Yes
(HE)_3_-Z_TfR#14_L27H_-biotin	66	Partly
(HE)_3_-Z_TfR#14_A9H_L27H_-biotin	62	Partly
(HE)_3_-Z_TfR#18_I11N_-biotin	52	Partly
(HE)_3_-Z_TfR#18_M14H_--biotin	62	Yes
(HE)_3_-Z_TfR#18_I11N_M14H_-biotin	63	Yes
(HE)_3_-Z_HER2_-biotin	62	Yes

**Table 2:** Estimated pH environment for Z_TfR_-FITC constructs incubated with bEnd.3 cells. Values are given as mean ± s.d. based on n = 3 samples.

Sample	Calculated pH Environment by Standard Curve	Endocytosis Classification
(HE)_3_-Z_HER2_-FITC	5.75 ± 0.02	Late/lysosomal
(HE)_3_-Z_TfR#14_A9H_-FITC	7.15 ± 0.31	Partial cell surface or vesicle
(HE)_3_-Z_TfR#14_L27H_-FITC	6.94 ± 0.02	Early
(HE)_3_-Z_TfR#14_A9H_L27H_-FITC	6.18 ± 0.04	Recycling
(HE)_3_-Z_TfR#18_I11N_-FITC	5.13 ± 0.02	Lysosome
(HE)_3_-Z_TfR#18_M14H_-FITC	6.56 ± 0.02	Early
(HE)_3_-Z_TfR#18_I11N_M14H_-FITC	5.16 ± 0.01	Lysosome

## Data Availability

Data is contained within the article and supplementary material.

## Supplementary Materials

The following supporting information can be downloaded at: https://www.mdpi.com/article/10.3390/ph16070956/s1, Figure S1: Phage selection procedure; Figure S2: Sequence alignment of clones, Table S1: Production of His_6_-Z_TfR_-ABD, Figure S3: Sodium Dodecyl Sulphate–Poly Acrylamide Gel (SDS-PAGE) of constructs His_6_-Z_TfR_-ABD, Table S2: Production of Z_TfR_-His_6_, Figure S4: SDS-PAGE of constructs Z_TfR_-His_6_, Figure S5: Circular dichroism (CD) of His_6_-Z_TfR_-ABD constructs, Table S3: Cellular binding of His_6_-Z_TfR_-ABD, Figure S6: Concentration dependent human cell binding, Table S4: Self-block of His_6_-Z_TfR_-ABD construct at cell binding, Figure S7: Histograms of competitive transferrin binding of His_6_-Z_TfR_-ABD constructs, Table S5: Transferrin competitive binding to His_6_-Z_TfR_-ABD, Figure S8: Secondary structure pH dependency, Figure S9: Surface plasmon resonance (SPR) sensorgram for TfR binding, Table S6: Kinetic data of SPR sensorgram, Figure S10: Single amino acid mutagenesis binding analysis, Table S7: Producibility of (HE)_3_-Z_TfR_-cys constructs, Figure S11: SDS-PAGE of production from (HE)_3_-Z_TfR_-cys constructs, Table S8: Biotinylation efficacy of (HE)_3_-Z_TfR_-cys constructs and thermal melting point, Figure S12: Cellular binding verification of (HE)_3_-Z_TfR_-FITC constructs, Figure S13: Cellular pH dependent binding verification of (HE)_3_-Z_TfR_-FITC constructs, Figure S14: Standard curve for pH dependent FITC signal, Table S9: Apparent permeability of (HE)_3_-Z_TfR_-FITC constructs.
