# Supplementary material for: Affibody Molecules Intended for Receptor-Mediated Transcytosis via the Transferrin Receptor
Source: Pharmaceuticals (Basel). 2023 Jul 3;16(7):956. doi: 10.3390/ph16070956 (PMC10383291; doi:10.3390/ph16070956)
Supplement: Supplementary file 1 [file pharmaceuticals-16-00956-s001.zip › pharmaceuticals-2449463-supplementary.pdf]

Supplementary material

Affibody Molecules Intended for Receptor-Mediated Transcytosis via the Transferrin Receptor

Linnea Charlotta Hjelm[1], Hanna Lindberg[1], Stefan Ståhl[1], and John Löfblom[1*]

Department of Protein Science, School of Engineering Sciences in Chemistry, Biotechnology and Health, KTH Royal Institute of Technology, 106 91 Stockholm, Sweden.

*Corresponding author. E-mail: lofblom@kth.se

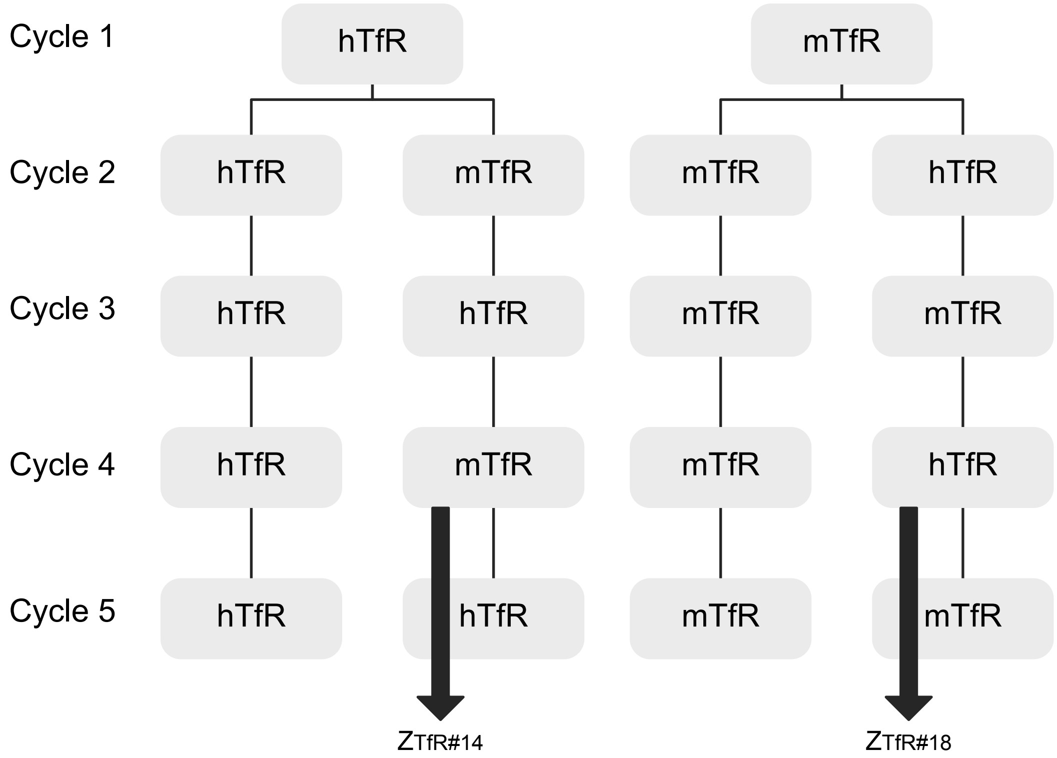

Figure S1. Phage selection procedure. Selection from phage library was performed in four tracks with different strategies. Clones from selection cycle 4 and 5 were analysed and characterised. $Z_{TfR\#14}$ and $Z_{TfR\#18}$ are derived from the fourth round of selections from the tracks with alternating murine and human TfR.

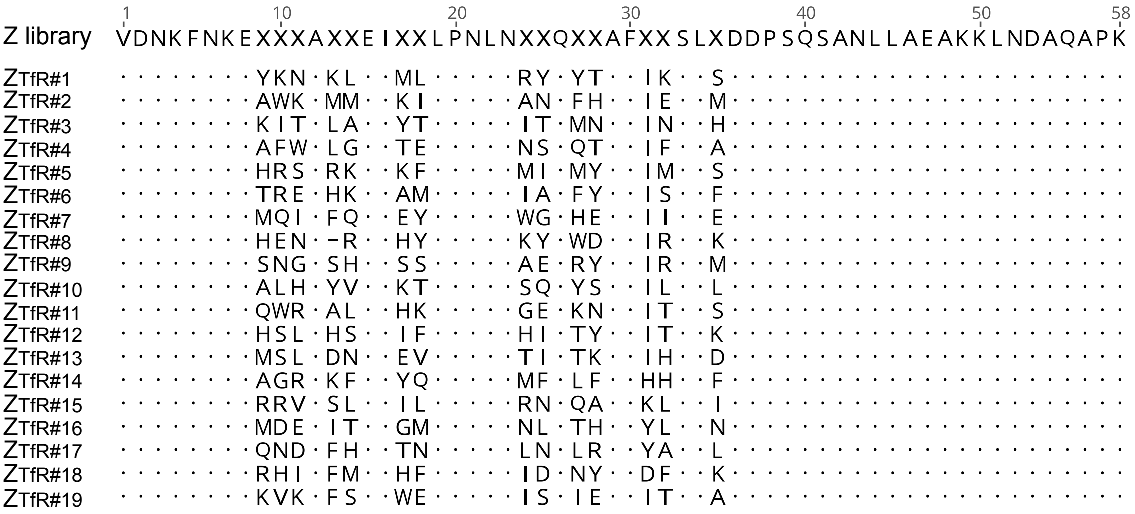

Figure S2. Sequence alignment of clones. Clones were identified as positive in phage ELISA and selected for characterisation. Aligned to reference sequence for the affibody library with randomised positions marked with "X".

Table S1. Production of His_6-Z_{TfR}-ABD. Producibility and molecular weight (Mw) of constructs validated by Maldi-TOF for [His_6-Z_{TfR}-ABD].

Construct	Amount protein [mg × 100 mL^{-1}]	Fold expression compared to Ztaq-ABD	Mw expected [Da]	Mw observed [Da]
**His_6-$Z_{TfR\#1}$-ABD**	0.91	0.49	14,489	14,481
**His_6-$Z_{TfR\#2}$-ABD**	0.86	0.46	14,418	14,413
**His_6-$Z_{TfR\#3}$-ABD**	2.28	1.21	14,301	14,296
**His_6-$Z_{TfR\#4}$-ABD**	1.58	0.84	14,253	14,245
**His_6-$Z_{TfR\#5}$-ABD**	0.39	0.21	14,496	14,226
**His_6-$Z_{TfR\#6}$-ABD**	1.60	0.86	14,382	14,396
**His_6-$Z_{TfR\#7}$-ABD**	2.68	1.43	14,491	14,480
**His_6-$Z_{TfR\#8}$-ABD**	1.41	0.75	14,513	14,497
**His_6-$Z_{TfR\#9}$-ABD**	2.62	1.40	14,263	14,266
**His_6-$Z_{TfR\#10}$-ABD**	1.98	1.06	14,304	14,312
**His_6-$Z_{TfR\#11}$-ABD**	2.08	1.11	14,336	14,338
**His_6-$Z_{TfR\#12}$-ABD**	0.71	0.38	14,365	14,352
**His_6-$Z_{TfR\#13}$-ABD**	4.31	2.29	14,284	14,276
**His_6-$Z_{TfR\#14}$-ABD**	0.62	0.33	14,497	14,517
**His_6-$Z_{TfR\#15}$-ABD**	0.30	0.16	14,348	14,325
**His_6-$Z_{TfR\#16}$-ABD**	1.16	0.62	14,320	14,326
**His_6-$Z_{TfR\#17}$-ABD**	0.49	0.26	14,387	14,387
**His_6-$Z_{TfR\#18}$-ABD**	0.87	0.47	14,551	14,540
**His_6-$Z_{TfR\#19}$-ABD**	3.76	2.0	14,319	14,311
**His_6-Ztaq-ABD**	1.88	n.a.	14,366	14,355

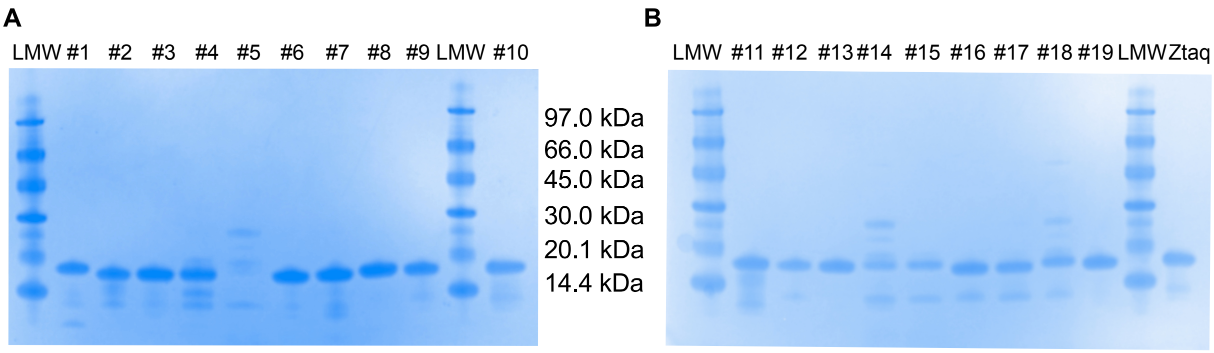

Figure S3. Sodium Dodecyl Sulphate–Poly Acrylamide Gel (SDS-PAGE) of constructs His_6-Z_{TfR}-ABD of 1.5 ug per lane for clones **(A)** #1–10 and **(B)** #11–19 together with Ztaq control. The expected weight of the constructs is approximately 14.5 kDa and is correlating well with the observed masses. Low molecular weight ladder (LMW) is included for comparison. A partial degradation product is seen for some constructs as masses below the expected weight. Clones with a low concentration show a variability in the intensity of the signal and could indicate an uncertainty in the concentration as $Z_{TfR\#5}$-ABD, $Z_{TfR\#14}$-ABD, and $Z_{TfR\#15}$-ABD.

Table S2. Production of Z_{TfR}-His_6. Cultivation and purification data for producibility of constructs in the Z_{TfR}-His_6 format. Molecular mass (Mw) is verified by Maldi-TOF. The mass spectroscopy showed a product with and without a methionine as visible in the SDS-PAGE, only non-methionine product peak is shown in table.

Construct	Amount protein [mg × 100 mL^{-1}]	Fold expression compared to Ztaq-ABD	Mw expected [Da]	Mw observed [Da]
**$Z_{TfR\#1}$-His_6**	0.93	0.06	7,662	7,671
**$Z_{TfR\#2}$-His_6**	4.13	0.26	7,740	7,733
**$Z_{TfR\#3}$-His_6**	7.40	0.47	7,474	7,488
**$Z_{TfR\#4}$-His_6**	5.79	0.37	7,574	7,574
**$Z_{TfR\#5}$-His_6**	0.61	0.04	7,818	7,810
**$Z_{TfR\#6}$-His_6**	2.73	0.17	7,555	7,566
**$Z_{TfR\#7}$-His_6**	1.04	0.07	7,664	7,678
**$Z_{TfR\#8}$-His_6**	4.14	0.27	7,686	n.d.
**$Z_{TfR\#9}$-His_6**	17.79	1.14	7,584	7,623
**$Z_{TfR\#10}$-His_6**	6.72	0.43	7,626	7,624
**$Z_{TfR\#11}$-His_6**	3.08	0.20	7,658	7,647
**$Z_{TfR\#12}$-His_6**	0.76	0.05	7,538	7,549
**$Z_{TfR\#13}$-His_6**	0.62	0.04	7,605	7,603
**$Z_{TfR\#14}$-His_6**	1.17	0.07	7,670	7,628
**$Z_{TfR\#15}$-His_6**	0.62	0.04	7,521	7,534
**$Z_{TfR\#16}$-His_6**	3.7	0.24	7,493	7,502
**$Z_{TfR\#17}$-His_6**	18.81	1.20	7,560	7,703
**$Z_{TfR\#18}$-His_6**	0.92	0.06	7,724	7,891
**$Z_{TfR\#19}$-His_6**	8.94	0.57	7,492	7,502
**Ztaq-His_6**	15.61	n.a.	6,062	n.d.

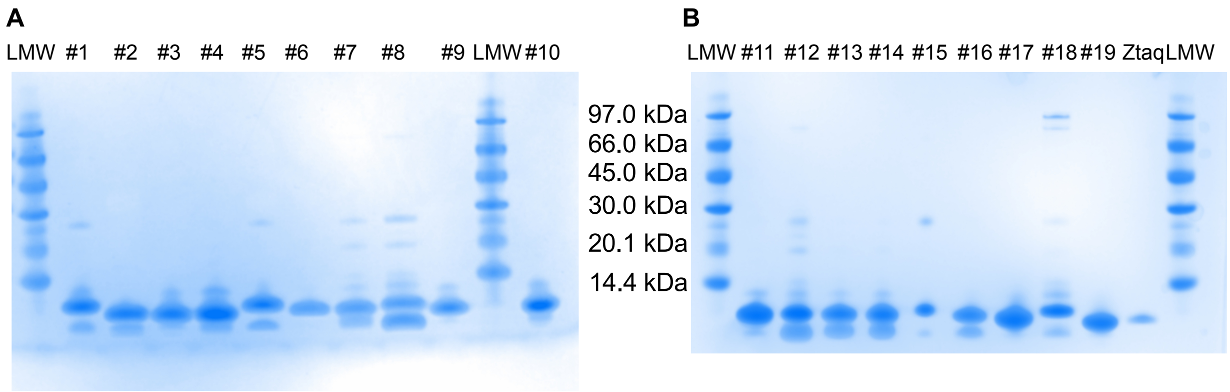

Figure S4. SDS-PAGE on Z_{TfR}-His$_6$ constructs **(A)** #1–10 and **(B)** #11–19 together with Ztaq control. Expected mass is approximately 7.5 kDa. Low molecular weight ladder (LMW) is included for comparison. For $Z_{TfR\#18}$ a contamination of likely a heat shock protein is seen in the 96 kDa range and some constructs show a partial degradation product below the expected mass of 7.5 kDa or the double production of the construct with and without methionine as shown in MS analysis.

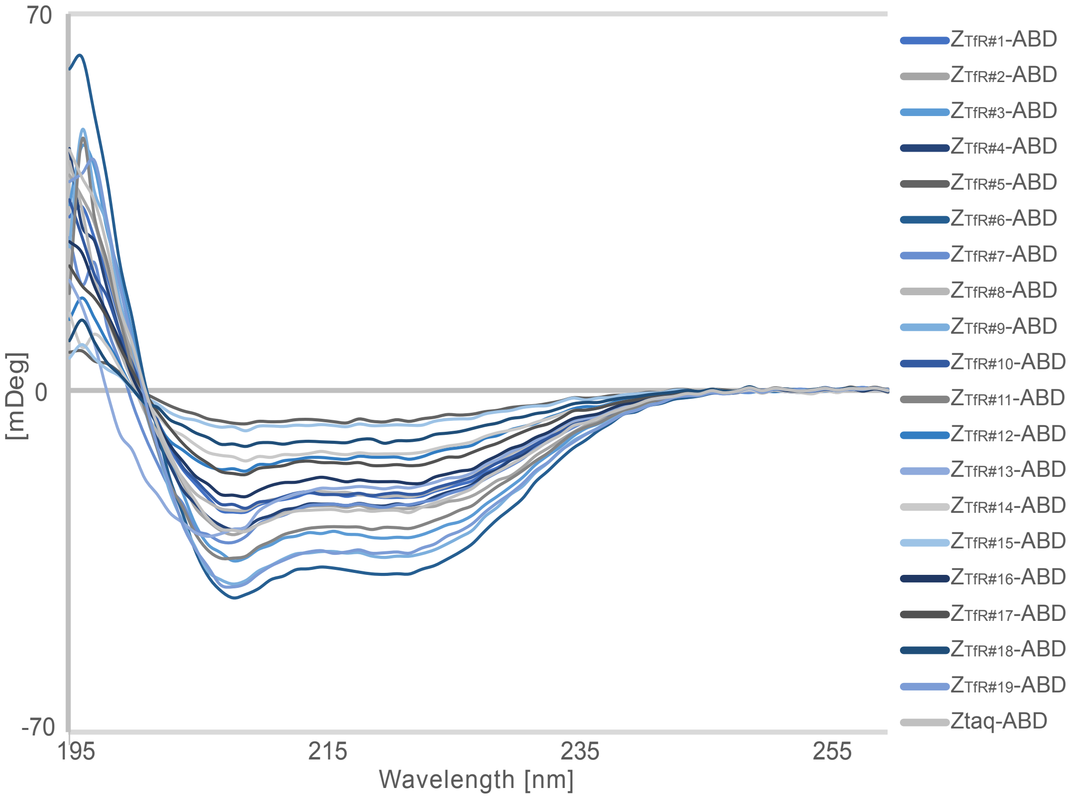

Figure S5. Circular dichroism (CD) for the Z_{TfR}-ABD constructs. Most proteins were analyzed at a concentration of 0.2 mg × mL^{-1}. Z_{TfR} constructs #5, #14, #15, and #17 did not have high enough concentration to be run at 0.2 mg × mL^{-1} and yield a lower signal.

Table S3. Cellular binding of His$_6$-Z$_{TfR}$-ABD. Mean fluorescent intensity (MFI) for cellular binding between the [His$_6$-Z$_{TfR}$-ABD] and TfR-expressing cells bEnd.3 and SK-OV-3. Normalised to the blank cells without any fluorophore added for each cell line. Controls with cells and only HSA-647 fluorophore, the control affibody Ztaq or mTfR specific scFv8D3-Z$_{SYM73}$-ABD are included. Detection of binding was done by HSA-647 measured at 640 nm laser and 660 nm BP filter.

	Average mean bEnd.3	Average mean SK-OV-3
Blank cells	1.00	1.00
HSA-647	1.72	1.72
Ztaq-ABD	2.20	2.16
scFv8D3-Z$_{SYM73}$-ABD	4.13	2.27
Z$_{TfR\#1}$-ABD	2.40	1.93
Z$_{TfR\#2}$-ABD	2.09	3.09
Z$_{TfR\#3}$-ABD	2.65	2.26
Z$_{TfR\#4}$-ABD	1.47	4.20
Z$_{TfR\#6}$-ABD	1.49	2.03
Z$_{TfR\#7}$-ABD	1.80	2.23
Z$_{TfR\#8}$-ABD	2.05	1.86
Z$_{TfR\#9}$-ABD	1.72	1.57
Z$_{TfR\#10}$-ABD	1.69	4.33
Z$_{TfR\#11}$-ABD	2.02	2.93
Z$_{TfR\#12}$-ABD	1.79	2.69
Z$_{TfR\#13}$-ABD	2.02	2.85
Z$_{TfR\#14}$-ABD	6.58	47.52
Z$_{TfR\#16}$-ABD	2.32	2.15
Z$_{TfR\#17}$-ABD	2.33	2.06
Z$_{TfR\#18}$-ABD	7.20	28.15
Z$_{TfR\#19}$-ABD	1.70	1.96

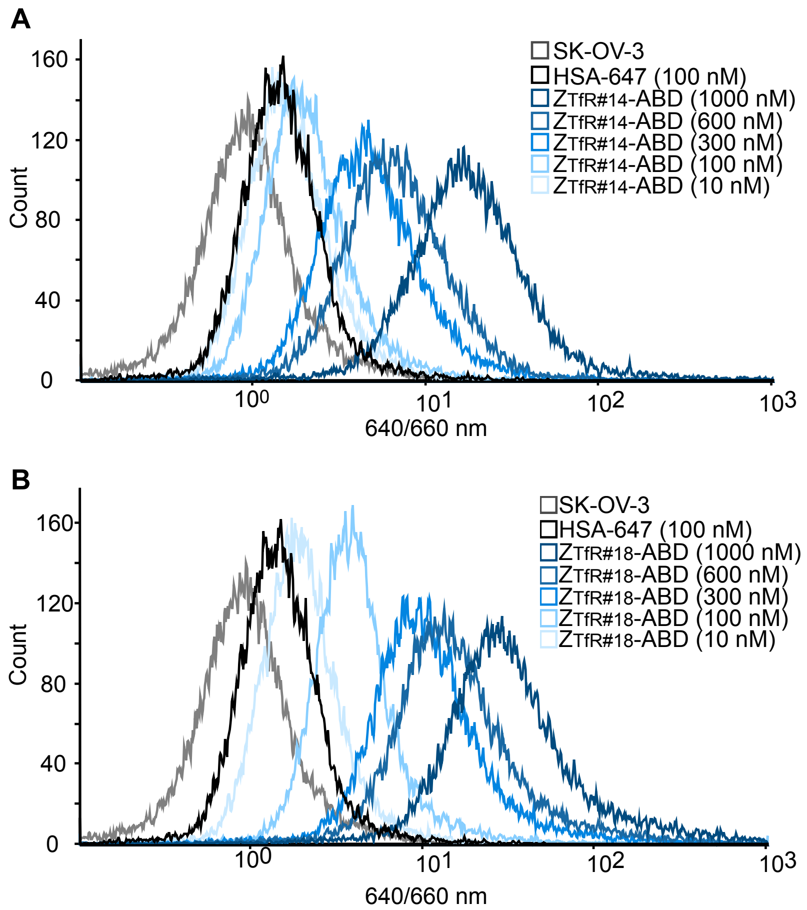

Figure S6. Concentration dependent human cell binding. Concentration-dependent binding of the constructs determined by flow cytometry for **(A)** $Z_{TfR\#14}$-ABD and **(B)** $Z_{TfR\#18}$-ABD in the range of 10–1000 nM (light to dark) detected by HSA-647 to the human expressing TfR cell line SK-OV-3. The y-axis shows counted cells and x-axis the fluorescent signal from HSA-647 measured at 640 nm laser and 660 nm BP filter. In total 20,000 cells were analysed per sample.

Table S4. Self-block of His_6-Z_{TfR}-ABD construct at cell binding. Flow cytometry data from self-block between His_6-Z_{TfR}-ABD and Z_{TfR}-His_6 constructs. Background signal from HSA is included. A shift is seen when 1:5 molar excess of the non-fluorescent clonality is added. In parenthesis the percentage of the signal blocked is shown. The constructs appear not to share epitope where at five times molar excess no change is seen in MFI signal.

Construct	Blocking 1:0 [MFI]	Blocking 1:1 [MFI] (% loss of signal)	Blocking 1:2 [MFI] (% loss of signal)	Blocking 1:5 [MFI] (% loss of signal)	Block ZTfR#18 1:5 [MFI]
**$Z_{TfR\#14}$-ABD**	15.6	13.06 (13%)	12.89 (17%)	7.16 (44%)	0%
**$Z_{TfR\#18}$-ABD**	8.19	8.27 (0%)	6.3 (23%)	5.55 (32%)	n.a.

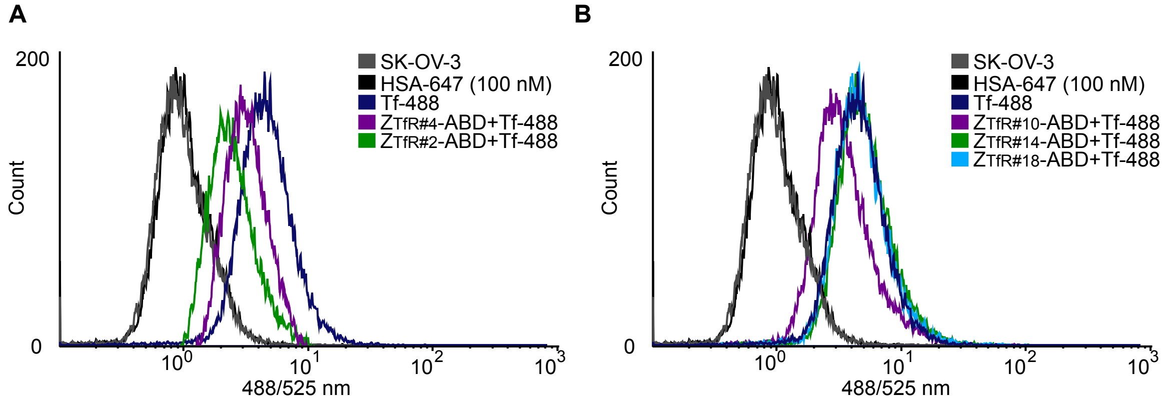

Figure S7. Histograms of competitive transferrin binding of His_6-Z_{TfR}-ABD constructs. Flow cytometry of SK-OV-3 cells. Fluorescence from cells corresponding to transferrin-AF488 (Tf-488)-binding. $Z_{TfR\#14}$-ABD or $Z_{TfR\#18}$-ABD co-incubated with different molar excess of Tf-488. Only cells are shown in grey and cells incubated with only secondary reagent are shown in black. Cells incubated with only Tf-488 are shown in dark blue. Cells incubated with affibody and Tf are shown in green (higher molar excess in darker shade). Tf-488 is detected at 488/525 nm laser and filter. **(A)** $Z_{TfR\#2}$-ABD and $Z_{TfR\#4}$-ABD co-incubated with Tf-488. **(B)** $Z_{TfR\#10}$-ABD, $Z_{TfR\#14}$-ABD and $Z_{TfR\#18}$-ABD co-incubated with Tf-488.

Table S5. Transferrin competitive binding to His_6-Z_{TfR}-ABD. Mean fluorescence intensity (MFI) from flow cytometry on SK-OV-3 cells co-incubated with Tf-488 and $Z_{TfR\#14}$-ABD, $Z_{TfR\#18}$-ABD and Z_{taq}-ABD (negative control). MFI 488/525 nm is corresponding to Tf-binding and MFI 640/660 nm is corresponding to Z_{TfR}-ABD-binding.

Z_{TfR} construct	Concentration Z_{TfR} [nM]	MFI 640/660 nm	Concentration Tf-488 [$\mu g \times mL^{-1}$]	MFI 488/525 nm
**n.a.**	0	2.037	0	0.944
**n.a.**	0	0.775	25	7.957
**$Z_{TfR\#14}$-ABD**	100	7.751	25	7.220
**$Z_{TfR\#14}$-ABD**	300	20.537	25	8.346
**$Z_{TfR\#14}$-ABD**	600	46.302	25	9.031
**$Z_{TfR\#18}$-ABD**	100	5.359	25	7.859
**$Z_{TfR\#18}$-ABD**	300	16.549	25	7.331
**$Z_{TfR\#18}$-ABD**	600	23.092	25	10.276
**Ztaq-ABD**	200	1.621	25	7.512
**Ztaq-ABD**	600	3.691	25	11.432

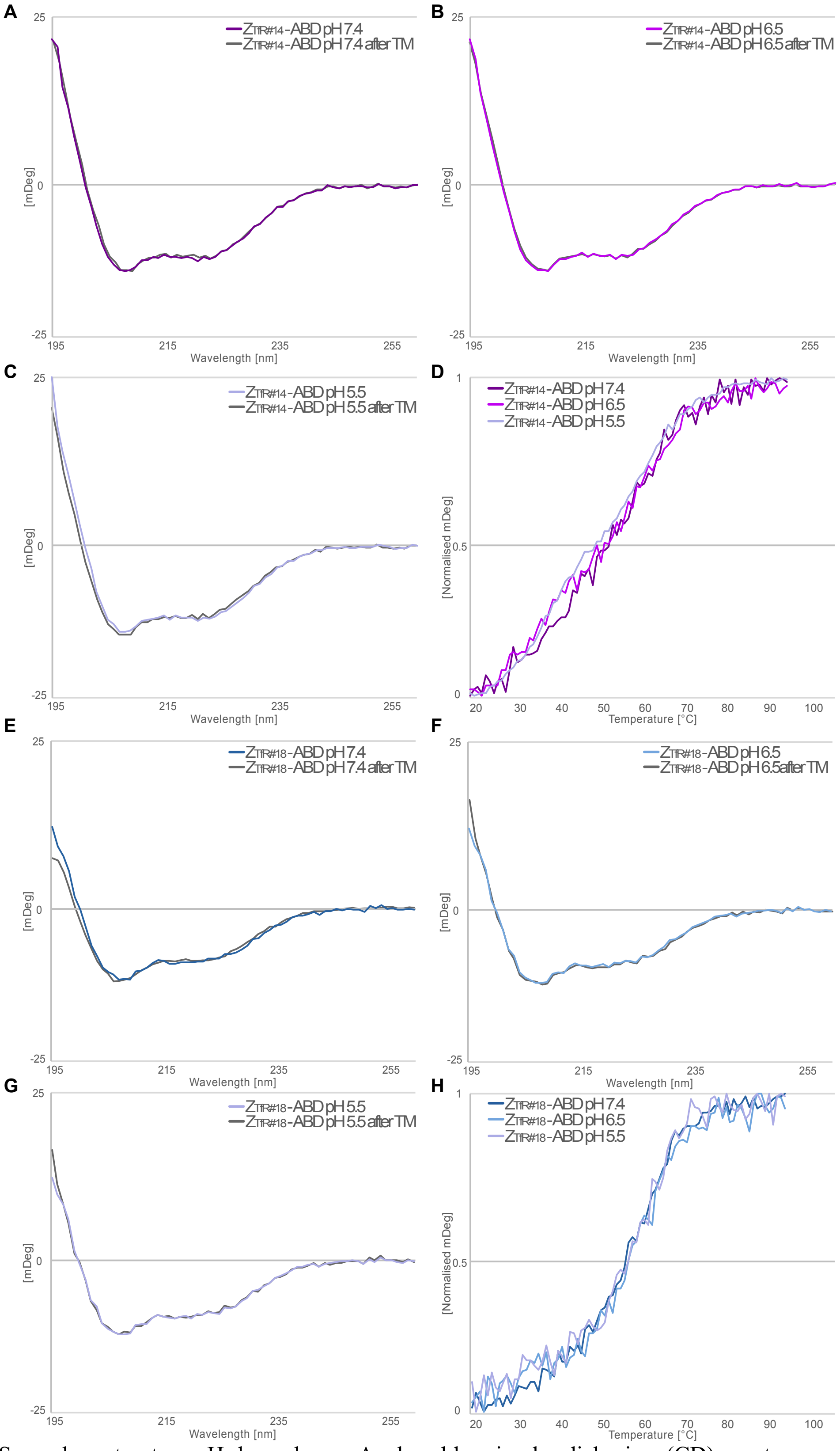

Figure S8. Secondary structure pH dependency. Analysed by circular dichroism (CD) spectroscopy on $Z_{TfR\#14}$-ABD and $Z_{TfR\#18}$-ABD **(A–C)** Secondary structure determined by CD between 195 and 260 nm for $Z_{TfR\#14}$-ABD at pH 5.5, 6.5 and 7.4 before and after heat-induced denaturation and refolding. **(D)** Measurement of thermal melting temperature for $Z_{TfR\#14}$-ABD at pH 5.5, 6.5 and 7.4. **(E–G)** Secondary structure determined by CD between 195 and 260 nm for $Z_{TfR\#18}$-ABD at pH 5.5, 6.5 and 7.4 before and after heat-induced denaturation and refolding. **(H)** Measurement of thermal melting temperature for $Z_{TfR\#18}$-ABD at pH 5.5, 6.5 and 7.4.

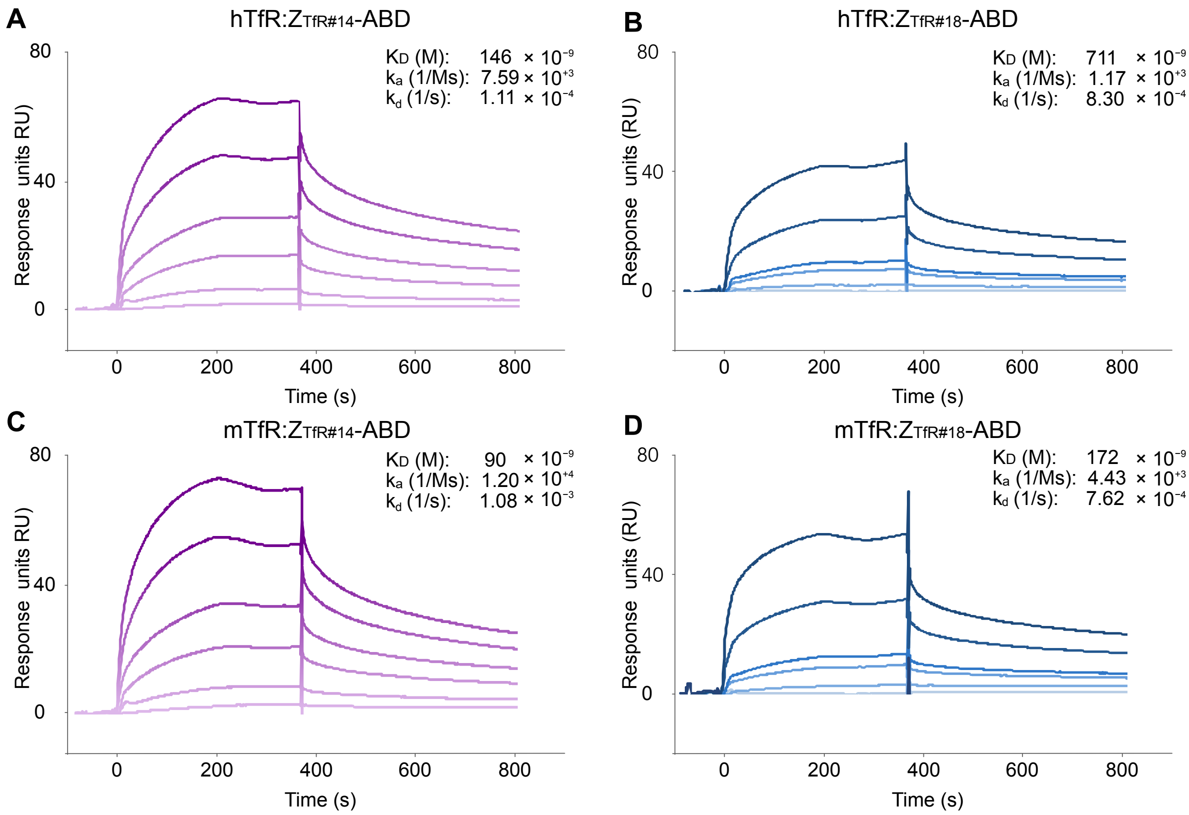

Figure S9. Surface plasmon resonance (SPR) sensorgram for TfR binding. SPR sensorgram for immobilised **(A–B)** human TfR and **(C–D)** murine TfR injected with 583–19.4 nM in a 1:1 dilution series $Z_{\text{TfR\#14}}$-ABD (purple) and $Z_{\text{TfR\#18}}$-ABD (blue). Kinetic constants and K_D are indicated in the figure and data from fitting to a 1:1 binding equation is shown in table S5.

Table S6. Kinetic data of SPR sensorgram. Association rate constant (k_a), dissociation rate constant (k_d), equilibrium dissociation constant (K_D), calculated RU max, mass transfer constant (tc), and Chi^2 value from fitting SPR data to a 1:1 binding equation. Affibody analytes are in ABD fused format.

**Ligand:analyte**	**k_a [$M^{-1}s^{-1}$]**	**k_d [s^{-1}]**	**K_D [M]**	**RU (max)**	**Mass transfer constant (tc)**	**Chi^2**
**hTfR:$Z_{\text{TfR\#14}}$**	7.59×10^3	1.11×10^{-3}	1.46×10^{-7}	56	1.06×10^8	3.54
**mTfR:$Z_{\text{TfR\#14}}$**	1.20×10^4	1.08×10^{-3}	9.00×10^{-8}	44	1.18×10^7	3.82
**hTfR:$Z_{\text{TfR\#18}}$**	1.17×10^3	8.30×10^{-4}	7.11×10^{-7}	212	6.44×10^{16}	2.06
**mTfR:$Z_{\text{TfR\#18}}$**	4.43×10^3	7.62×10^{-4}	1.72×10^{-7}	44	1.59×10^{10}	2.77

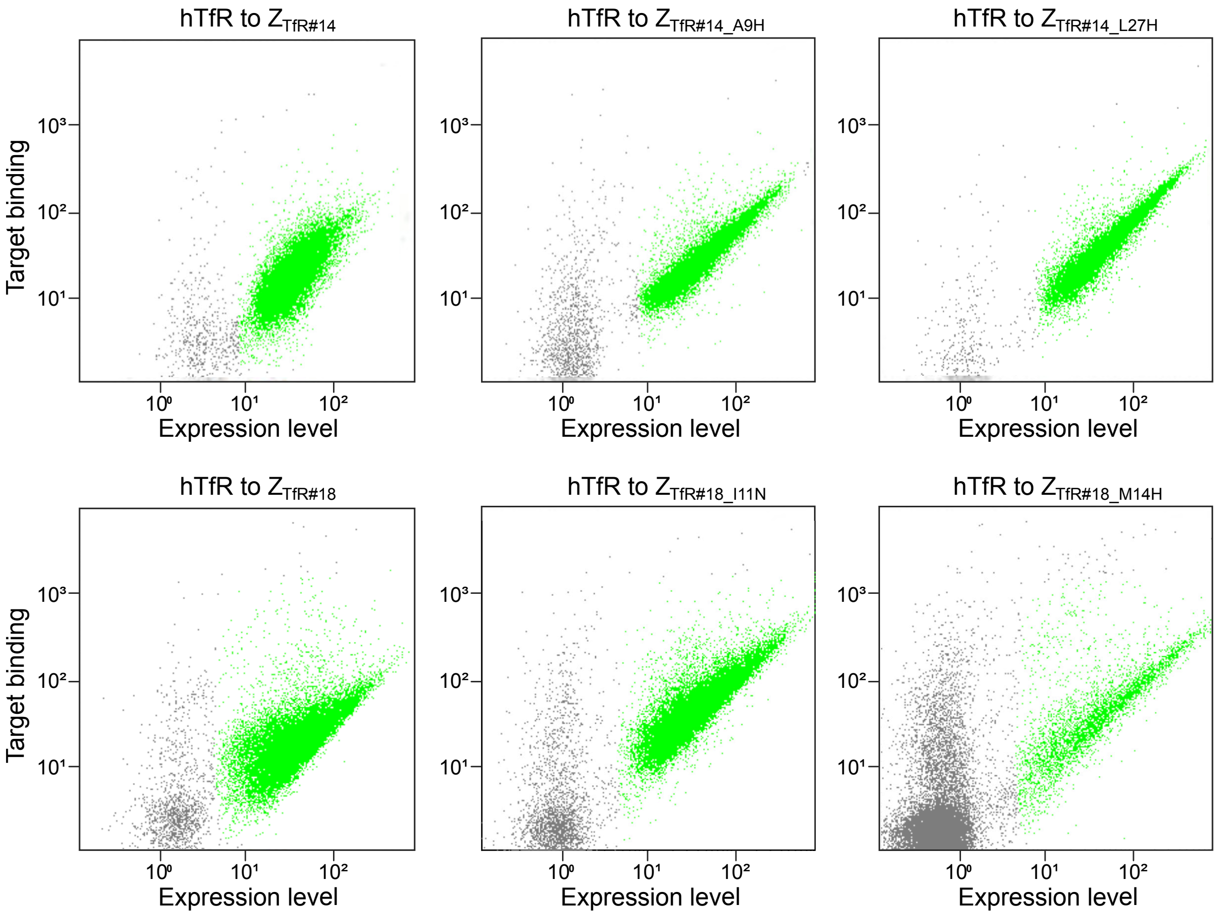

Figure S10. Single amino acid mutagenesis binding analysis. Flow cytometry on *E. coli* displaying original and mutated variants of $Z_{TfR\#14}$, and $Z_{TfR\#18}$ in fusion to an albumin-binding domain (ABD). Human TfR-binding on y-axis and surface expression level measured by binding to fluorescently labelled HSA on x-axis. **(A–C)** *E. coli* displaying $Z_{TfR\#14}$, $Z_{TfR\#14\_A9H}$, and $Z_{TfR\#14\_L27H}$ incubated with 100 nM hTfR. **(D–F)** *E. coli* displaying $Z_{TfR\#18}$, $Z_{TfR\#18\_I11H}$, and $Z_{TfR\#18\_M14H}$ incubated with 75 nM hTfR. Green includes HSA-positive ABD-expressing cell population.

Table S7. Producibility of $(HE)_3$-Z_{TfR}-cys constructs. Yield for $(HE)_3$-Z_{TfR}-cys constructs compared to the yield for the original $Z_{TfR\#14}$ or $Z_{TfR\#18}$ constructs in Z_{TfR}-His_6 format. Mw is verified by Maldi-TOF.

Clone	Yield [mg × 100 mL^{-1}]	Yield compared to original clonality	Mw expected [Da]	Mw observed [Da]
**$(HE)_3$-$Z_{TfR\#14\_A9H}$-cys**	0.98	0.8	7,722	7,721
**$(HE)_3$-$Z_{TfR\#14\_L27H}$-cys**	2.60	2.2	7,680	7,679
**$(HE)_3$-$Z_{TfR\#14\_A9H\_L27H}$-cys**	2.02	1.7	7,746	7,746
**$(HE)_3$-$Z_{TfR\#18\_I11N}$-cys**	3.45	3.8	7,711	7,710
**$(HE)_3$-$Z_{TfR\#18\_M14H}$-cys**	0.95	1.0	7,716	7,715
**$(HE)_3$-$Z_{TfR\#18\_I11N\_M14H}$-cys**	1.31	1.4	7,716	7,716
**$(HE)_3$-Z_{Her2}-cys**	10.83	n.a.	7,739	7,738

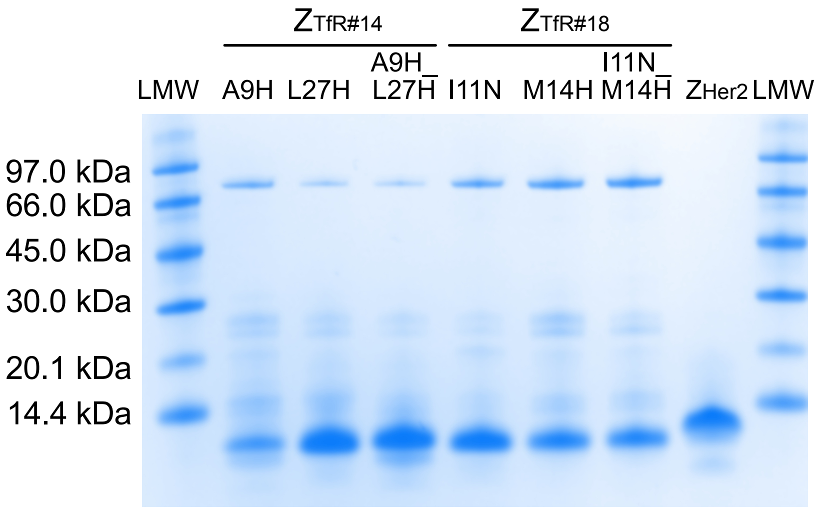

Figure S11. SDS-PAGE of production from $(HE)_3$-Z_{TfR}-cys constructs. SDS-PAGE for 1.5 µg per lane of non-conjugated $(HE)_3$-Z_{TfR}-cys. Partial dimerization of the constructs are observed around 14 kDa. A contaminant of co-purified host cell protein at approximately 90 kDa is visible.

Table S8. Biotinylation efficacy of $(HE)_3$-Z_{TfR}-cys constructs and thermal melting point. Degree of biotinylation, thermal melting point (T_m) and refolding for biotinylated single amino acid mutants, $Z_{TfR\#14\_A9H}$, $Z_{TfR\#14\_L27H}$, $Z_{TfR\#14\_A9H\_L27H}$, $Z_{TfR\#18\_I11N}$, $Z_{TfR\#18\_M14H}$, $Z_{TfR\#18I\_11N\_M14H}$, and Z_{HER2}.

Clone	Percentage biotinylated	T_m [°C]	Refolding capability
**$(HE)_3$-$Z_{TfR\#14\_A9H}$-biotin**	>80%	64	Yes
**$(HE)_3$-$Z_{TfR\#14\_L27H}$-biotin**	100%	66	Partly
**$(HE)_3$-$Z_{TfR\#14\_A9H\_L27H}$-biotin**	>80%	62	Partly
**$(HE)_3$-$Z_{TfR\#18\_I11N}$-biotin**	>60%	52	Partly
**$(HE)_3$-$Z_{TfR\#18\_M14H}$-biotin**	>60%	62	Yes
**$(HE)_3$-$Z_{TfR\#18\_I11N\_M14H}$-biotin**	>50%	63	Yes
**$(HE)_3$-Z_{HER2}-biotin**	>70%	62	Yes

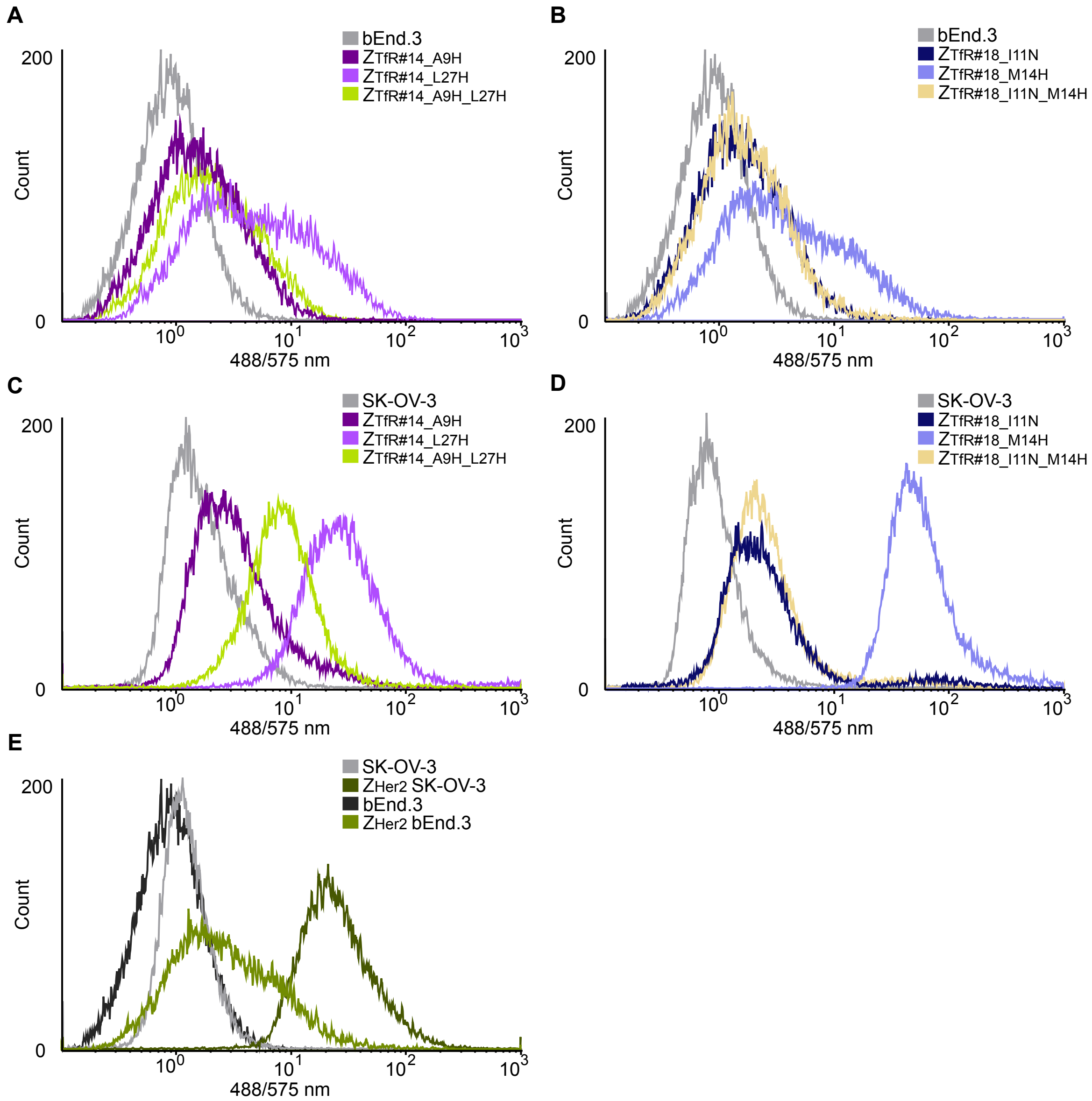

Figure S12. Cellular binding verification of $(HE)_3$-Z_{TfR}-FITC constructs. Flow cytometry analysis of FITC labelled $Z_{TfR\#14\_A9H}$, $Z_{TfR\#14\_L27H}$, $Z_{TfR\#14\_A9H\_L27H}$, $Z_{TfR\#18\_I11N}$, $Z_{TfR\#18\_M14H}$, $Z_{TfR\#18I\_11N\_M14H}$, and Z_{HER2}. **A–B)** bEnd.3 cells and **C–D)** SK-OV-3 cells incubated with 1 µM Z_{TfR}. **E)** bEnd.3 and SK-OV-3 cells incubated with HER2-specific control affibody (Z_{HER2}). bEnd.3 cells (dark grey), bEnd.3 cells with Z_{Her2} (light green), SK-OV-3 cells (light grey) and SK-OV-3 cells with Z_{Her2} (dark green).

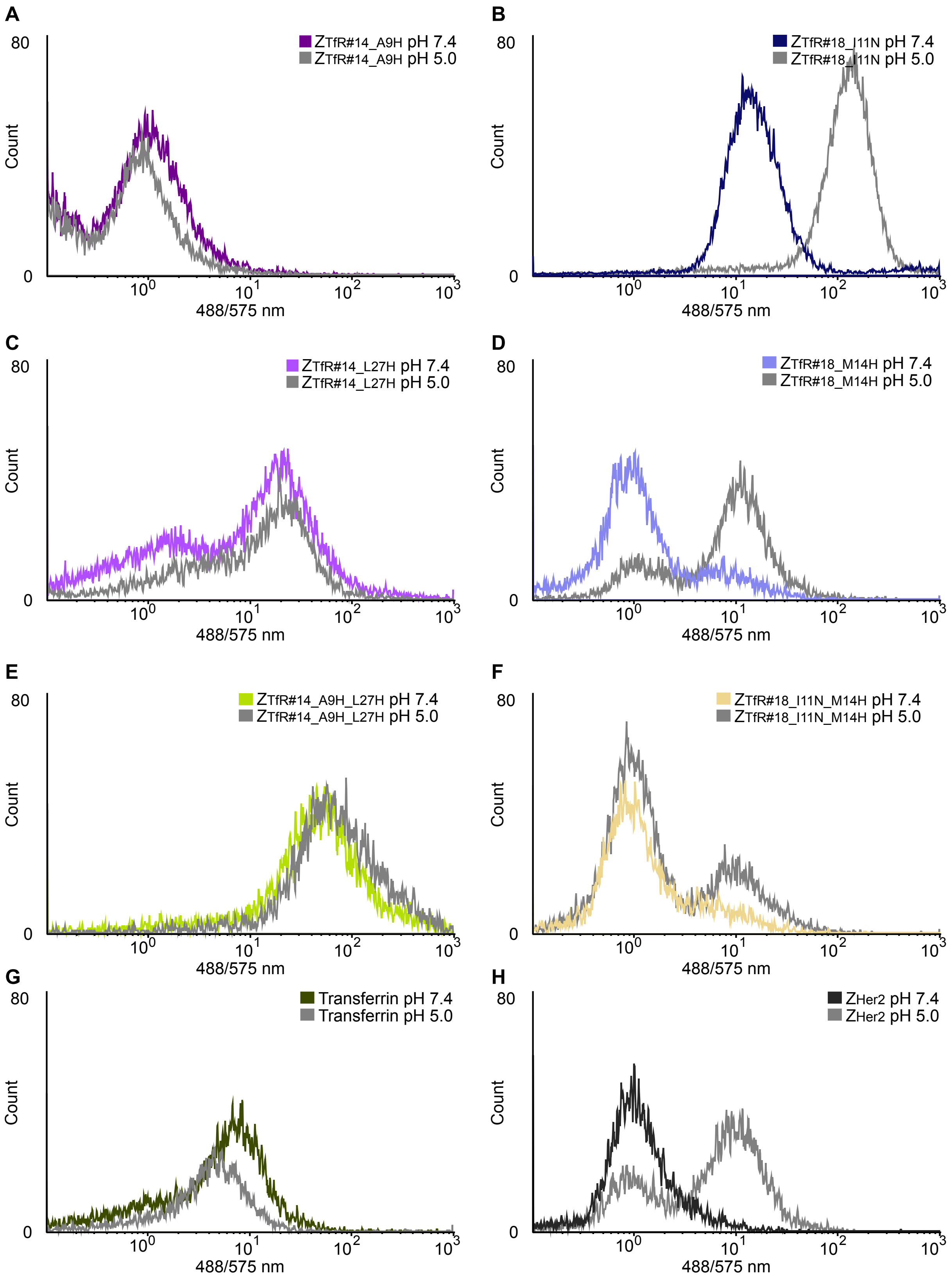

Figure S13. Cellular pH dependent binding verification of $(HE)_3$-Z_{TfR}-FITC constructs. Flow cytometry on SK-OV-3 cells. Cells were incubated with Z_{TfR}, Z_{HER2} or Tf-488 at pH 7.4 before cells were washed and divided into two samples for dissociation at pH 7.4 or 5.0 for 30 min. The samples are finally washed in pH 7.4 and analysed in the flow cytometer. The signal obtained after incubation at different pH for **(A)** $Z_{TfR\#14\_A9H}$, **(B)** $Z_{TfR\#18\_I11N}$, **(C)** $Z_{TfR\#14\_L27H}$, **(D)** $Z_{TfR\#18\_M14H}$, **(E)** $Z_{TfR\#14\_A9H\_L27H}$, **(F)** $Z_{TfR\#18\_I11N\_M14H}$, **(G)** Tf-488, and **(H)** Z_{HER2}.

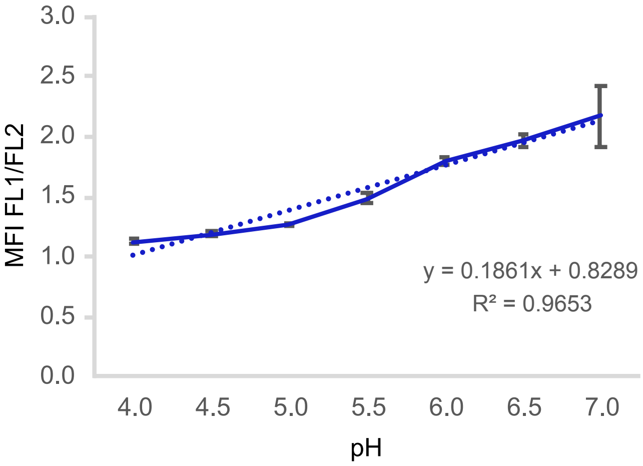

Figure S14. Standard curve for pH dependent FITC signal. The standard curve from dextran-FITC mean fluorescent intensity (MFI) for FL1/FL2 ratio. The curve had a linear fit (dashed line) of 0.965 in regression value. Error bars for the triplicate standard points are given for each pH step.

Table S9. Apparent permeability of $(HE)_3$-Z_{TfR}-FITC constructs. Transcytosis assay with the Z_{TfR}-FITC labelled proteins over bEnd.3 cell membrane on recombinant silk membrane. All samples were analyzed in triplicate. The two-sided student t-test is used for comparing apparent permeability (p_{app}) between affibody and the internal control (IgG2a) as membranes might differ in tightness.

Sample	p_{app} for Z	p_{app} for negative control	Recovery [%]	p-value
**$(HE)_3$-$Z_{TfR\#14\_A9H}$ -FITC**	$5.23 \times 10^{-6} \pm 0.88 \times 10^{-6}$	$6.17 \times 10^{-6} \pm 1.59 \times 10^{-6}$	95	0.657
**$(HE)_3$-$Z_{TfR\#14\_L27H}$ -FITC**	$1.07 \times 10^{-5} \pm 0.23 \times 10^{-5}$	$4.44 \times 10^{-6} \pm 2.55 \times 10^{-6}$	113	0.007
**$(HE)_3$-$Z_{TfR\#14\_A9H\_L27H}$ -FITC**	$5.08 \times 10^{-6} \pm 0.40 \times 10^{-6}$	$1.01 \times 10^{-6} \pm 2.26 \times 10^{-6}$	102	0.0000001
**$(HE)_3$-$Z_{TfR\#18\_I11N}$ -FITC**	$1.11 \times 10^{-5} \pm 0.23 \times 10^{-5}$	$4.68 \times 10^{-6} \pm 2.34 \times 10^{-6}$	117	0.050
**$(HE)_3$-$Z_{TfR\#18\_M14H}$ -FITC**	$5.30 \times 10^{-6} \pm 1.07 \times 10^{-6}$	$2.92 \times 10^{-6} \pm 0.85 \times 10^{-6}$	95	0.023
**$(HE)_3$-$Z_{TfR\#18\_I11N\_M14H}$ -FITC**	$1.04 \times 10^{-5} \pm 0.27 \times 10^{-5}$	$3.22 \times 10^{-6} \pm 1.22 \times 10^{-6}$	86	0.006
**$(HE)_3$-Z_{HER2}-FITC**	$4.14 \times 10^{-6} \pm 2.04 \times 10^{-6}$	$2.36 \times 10^{-6} \pm 1.69 \times 10^{-6}$	90	0.289
